# SPRTN protease-cleaved MRE11 decreases DNA repair and radiosensitises cancer cells

**DOI:** 10.1038/s41419-021-03437-w

**Published:** 2021-02-08

**Authors:** Juri Na, Joseph A. Newman, Chee Kin Then, Junetha Syed, Iolanda Vendrell, Ignacio Torrecilla, Sophie Ellermann, Kristijan Ramadan, Roman Fischer, Anne E. Kiltie

**Affiliations:** 1 MRC Oxford Institute for Radiation Oncology, Department of Oncology, University of Oxford, Oxford, OX3 7DQ UK; 2 Centre for Medicines Discovery, University of Oxford, Oxford, UK; 3 Target Discovery Institute, Nuffield Department of Medicine, University of Oxford, Oxford, UK

**Keywords:** DNA damage and repair, Urogenital diseases

## Abstract

The human MRE11/RAD50/NBS1 (MRN) complex plays a crucial role in sensing and repairing DNA DSB. MRE11 possesses dual 3′−5′ exonuclease and endonuclease activity and forms the core of the multifunctional MRN complex. We previously identified a C-terminally truncated form of MRE11 (TR-MRE11) associated with post-translational MRE11 degradation. Here we identified SPRTN as the essential protease for the formation of TR-MRE11 and characterised the role of this MRE11 form in its DNA damage response (DDR). Using tandem mass spectrometry and site-directed mutagenesis, the SPRTN-dependent cleavage site for MRE11 was identified between 559 and 580 amino acids. Despite the intact interaction of TR-MRE11 with its constitutive core complex proteins RAD50 and NBS1, both nuclease activities of truncated MRE11 were dramatically reduced due to its deficient binding to DNA. Furthermore, lack of the MRE11 C-terminal decreased HR repair efficiency, very likely due to abolished recruitment of TR-MRE11 to the sites of DNA damage, which consequently led to increased cellular radiosensitivity. The presence of this DNA repair-defective TR-MRE11 could explain our previous finding that the high MRE11 protein expression by immunohistochemistry correlates with improved survival following radical radiotherapy in bladder cancer patients.

## Introduction

MRE11 is a component of the conserved MRE11–RAD50–NBS1 (MRN) complex, a key player in the early stages of the DNA damage response (DDR). MRN is responsible for the recognition, repair, and signalling of DNA double-strand breaks (DSB)^1,2^ and is required for homologous recombination (HR), classical non-homologous end-joining (C-NHEJ) and alternative non-homologous end-joining (A-NHEJ) pathways in detection and signalling of DSBs^3^. Its effects are mediated via its 3′−5′ exonuclease and single-stranded (ss) and DNA hairpin endonuclease activities^4–7^. Absence of any of its components leads to embryonic lethality in mammals^4^. The 3′−5′ exo- and endonuclease activities of MRE11 have specialised roles in HR repair and in supporting ataxia-telangiectasia-mutated and Rad3- related (ATR) kinase activation^8,9^. Abrogation of MRE11 nuclease activity causes a striking array of phenotypes, indistinguishable from the absence of MRN, including early embryonic lethality and marked genomic instability^2,9^. Inherited MRE11 mutations cause symptoms very similar to the disease ataxia telangiectasia^10–12^.

We previously identified a C-terminally truncated form of MRE11 (TR-MRE11) which is generated post-translationally^13^. Here we investigate the cleavage site of TR-MRE11, its effects on DNA damage repair following ionising radiation and the likely associated protease. Having identified the putative cleavage site, we successfully established stable TR-MRE11 cell lines and produced recombinant truncated protein. TR-MRE11 lacked nuclease activity and DNA binding efficiency whilst still interacting efficiently with RAD50 and NBS1, thus likely maintaining an intact MRN complex structure lacking the MRE11 C-terminus. Cells expressing TR-MRE11 had deficient DNA damage repair function. Finally, we identified that the metalloprotease SPRTN as the key protease that induces TR-MRE11 accumulation.

## Materials and methods

### Cell culture

293T cells were obtained from ATCC, Manassas, VA, in 2011, and maintained in high glucose DMEM (Gibco-Thermo Fisher Scientific, CA) supplemented with 10% FBS (Thermo Fisher, Waltham, CA) without antibiotics. The cell lines RT112 and VM-CUB1 were purchased from the German Collection of Microorganisms and Cell Cultures (DSMZ), Braunschweig, Germany, and maintained in RPMI medium (MilliporeSigma, Burlington, MA) or DMEM medium (Gibco, Thermo Fisher Scientific, Waltham, MA), respectively supplemented with 10% FBS without antibiotics. Cells were maintained at 37 °C and 5% CO_2_ in a humidified incubator. 293T cells were confirmed to be mycoplasma-negative by PCR in January 2019. RT112 wildtype, RT112-MRE11 KD, and RT112-full length MRE11 were confirmed to be mycoplasma negative by PCR in September 2018. VM-CUB1 wildtype, VM-CUB1-MRE11 KD, and VM-CUB1-full length MRE11 were confirmed by MycoAlert (Lonza, Basel, Switzerland) in February 2020. Cell lines were used up to 30 passages from original stock, including the transfection and selection procedures.

### Protein digestion, LC-MS/MS and data analysis of MRE11 pull down

RT112 cells (2 × 10^7^ cells per dish) were plated in five 15 cm dishes and harvested 2 days later. Total protein concentration was determined by BCA assay (Thermo Fisher Scientific, Waltham, MA). For immunoprecipitations, 10 mg lysate was precleared with protein A/G dynabeads (Thermo Fisher Scientific, Waltham, MA) at 4 °C for 60 min. Lysates were incubated overnight with anti-MRE11 antibody (ab214) then added to protein A/G beads (40 µl) at 4 °C for 2 h. Beads were washed six times in PBS and binding proteins eluted by boiling for 10 min at 95 °C in 40 µl Laemmli buffer before running on an 4–20% SDS-PAGE gel. After instant blue staining, bands corresponding to FL- or TR-MRE11 were excised from the gel, cut into small pieces and subjected to an in-gel-digest process^14^. In brief, gel bands were destained, incubated with 10 mM DTT and 50 mM iodacetamide before incubating them with 60 ng of trypsin or elastase overnight at 37 °C. Peptides were extracted and dried down.

Dried peptides were reconstituted in 98% LC-MS/MS water, 2% acetonitrile and 0.1% TFA. Fifty to fifty-eight percent of the tryptic or elastase peptides were analysed over 1 h gradient from 2 to 35% acetonitrile in 5% dimethyl sulfoxide, 0.1% formic acid (at 250 nl/min) using a Dionex Ultimate 3000 UPLC connected to an Orbitrap Fusion Lumos trihybrid operated in data-dependent acquisition mode (both instruments from Thermo). Data were acquired with the universal method as described previously^15^. Briefly, full MS scans were acquired in the Orbitrap at 120 k resolution over a *m/z* range 400–1500, AGC target of 4e5 and S-lens RF of 30. Fragment ion spectra (MS/MS) were acquired in the Orbitrap (15 K resolution) with a Quad isolation window of 1.2, AGC target of 5e4 and a maximum injection time of 40 ms, with HCD activation and 28% collision energy.

MASCOT (vs2.4, Matrix Science) was used for peptide and protein identification. Data were searched against the human SwissProt database (downloaded 2017_08 for TR MRE11 and 2017_07 for FL MRE11). MASCOT results were reported after applying an ion score cut off of 20 and a 1% FDR above identity or homology threshold.

### Protein purification

FL-MRE11 and TR-MRE11 constructs were cloned into pNIC28-Bsa4 and SUMO2-LIC vectors respectively by LIC cleavage. Proteins were expressed in *E. coli* BL21(DE3)-R3-pRARE in Terrific Broth and were induced using IPTG (0.1 mM) at 18 °C (OD point 2–3). Cells were harvested by centrifugation. For purification, cell pellets were thawed and resuspended in buffer A (50 mM HEPES pH 7.5, 500 mM NaCl, 5% glycerol, 10 mM imidazole, 0.5 mM Tris (2-carboxyethyl) phosphene (TCEP)). Cells were lysed using sonication and cell debris pelleted by centrifugation. Lysates were applied to a Ni-NTA IMAC gravity flow column (Qiagen, Hilden, Germany), washed with two column volumes of wash buffer (buffer A supplemented with 45 mM imidazole), and eluted with the addition of 300 mM imidazole in buffer A. For FL-MRE11, the elution fraction was immediately concentrated, and gel filtration applied using a HiLoad 16/60 Superdex 200 column (GE health care, Chicago, IL). Fractions containing FL-MRE11 were harvested and cleaved with the addition of 1:20 mass ratio of TEV protease. For TR-MRE11, the purification tag was cleaved with the addition of 1:20 mass ratio of SUMO protease during overnight dialysis into buffer B (20 mM HEPES, pH 7.5, 500 mM NaCl, 5% glycerol, 0.5 mM TCEP). SUMO was removed by IMAC column rebinding and final protein purification was performed by size exclusion chromatography using a HiLoad 16/60 Superdex 200 column in buffer B at 1 ml/min in buffer B. Protein concentrations were determined by measurement at 280 nm (Nanodrop) using the calculated molecular mass and extinction coefficients, and intact masses were confirmed by ESI-MS. Coomassie stained gels of all constructs used in this study are shown in Fig. S4.

### ESI-MS mass spectrometry

Thirty microlitre protein samples at 0.02 mg/ml in 0.1% formic acid were injected onto a 4.6 × 50 mm Zorbax 5-μm 300SB-C3 column (AG883995-909; Agilent, Santa Clara, CA) and resolved by reversed-phase chromatography at 40 °C. The solvent system was 0.1% formic acid in double distilled H_2_O (buffer A) and 0.1% formic acid in methanol (buffer B), with 1 min at 5% buffer B and then a linear gradient of 5–95% buffer B over 6 min at 0.5 ml/min. Protein intact mass was determined using an MSD-TOF electrospray ionization orthogonal time-of-flight mass spectrometer (Agilent Technologies, Palo Alto, CA) operated in positive ion mode.

### Fluorescence polarization

For the DNA binding assay, oligonucleotides were chemically synthesized and purified by HPLC (Eurofins Scientific, Luxembourg). The oligonucleotide sequences were: TP423 (labelled with FITC at 5′): 5′-CTG CAG GGT TTT TGT TCC AGT CTG TAG CAC TGT GTA AGA CAG GCC AGA TC-3′; and TP424: 5′-CAC AGT GCT ACA GAC TGG AAC AAA AAC CCT GCA GTA CTC TAC TCA TCT C-3′. TP423 (labelled with FITC at 5′). TP424 oligonucleotides were mixed at 50 µM and heated to 96 °C in a heat block and were allowed to cool down slowly over 2 h to generate the 3′ overhanging DNA duplexes probes. The 3′ overhanging DNA duplexes (10 nM) were subsequently incubated with either purified FL-MRE11 or TR-MRE11 (10 nmol/L) proteins in the binding buffer (10 mM Tris at pH 8.0, 25 mM NaCl, 0.1% Tween20, 2 mM DTT) and fluorescence polarization measured immediately by a PHERAstar FSX (BMG Labtech, Aylesbury, UK) plate reader with excitation at 485 nm and emission at 520 nm. The FP values were plotted against the log of the protein concentrations, and the apparent dissociation constants were calculated from fitting the curves with a sigmoidal four-parameter logistic binding model.

### Nuclease assay

The substrate for the nuclease assay was prepared as detailed above using TP423 (labelled with FITC at 5’) and TP424. Reactions (20 µl) contained 100 nM DNA in HEPES buffer (25 mM HEPES at pH 7.5, 20 mM KCl, 0.2% Tween-20, 2 mM DTT, and 5 mM MnCl_2_). After incubation for 5 min at 37 °C, the indicated amounts of FL-MRE11 and TR-MRE11 were added, and incubation was continued for 2 h at 37 °C. Reaction products were run for 2 hr at 200 V on a 14% polyacrylamide gel with 8 M urea in 1× TBE. Gels were visualised at 488 nm using ChemiDoc (BioRad, Hercules, CA).

### Transfection

RT112-MRE11 KD cells and VM-CUB1-MRE11 KD cells were generated using psilencer-2.1-U6 neo (#5764; Thermo Fisher, Waltham, MA), as per the manufacturer’s recommendations. The shMRE11 sequences were as follows: shMRE11 forward; 5′-GAT CCG AAC CTG GTC CCA GAG GAG TTC AAG AGA CTC CTC TGG GAC CAG GTT CTT TTT TGG AAA-3′; and shMRE11 reverse; 5′-AGC TTT TCC AAA AAA GAA CCT GGT CCC AGA GGA GTC TCT TGA ACT CCT CTG GGA CCA GGT TCG-3′. After 3 days of transfection, 500 µg/ml of G418 was added for selection with complete media (RPMI or DMEM medium supplemented with 10% FBS for RT112 and VM-CUB1, respectively).

A lentiviral system was used to rescue full length MRE11 or truncated MRE11 expression. 293T cells were transfected in 10 cm dishes seeded at 5 × 10^6^ cells per dish, and 9 µg of either pLenti-full length MRE11 or pLenti-truncated MRE11, 4.5 µg of psPAX2 (#12260; Addgene, Watertown, MA), and 4.5 µg of pMD2.G (#12259; Addgene, Watertown, MA) were delivered to each dish with Lipofectamine 3000 (Thermo Fisher Scientific, Waltham, MA) in Opti-MEM (Thermo Fisher, Waltham, MA), according to the manufacturer’s instructions. The generated lentiviral particles were collected 48 hr following transfection and filtered through a 0.45 µm syringe filter (SLHV033RS; Millipore, Burlington, MA). RT112-MRE11 KD and VM-CUB1-MRE11 KD cells were infected with the filtered lentiviral supernatant using 8 µg/ml of polybrene. After 3 days of infection, 5 µg/ml of puromycin was added for selection with complete media.

### Ionising radiation

RT112-wildtype, VM-CUB1-wildtype, and MRE11-mutated stable cell lines were collected or fixed at various time point after various dose of Cs-137 ionising radiation (dose rate: 1.2 Gy/min) using a GSR-D1 irradiator (Gamma Services, Surrey, UK).

### Vectors and site-directed mutagenesis

MRE11 sequences were inserted into the vector pLenti-puro-CMV (P100022; Vigene, Rockville, MD). A QuickChange II XL Site-Directed Mutagenesis Kit (#200521; Agilent, Santa Clara, CA) and XL10-Gold ultracompetent cells were used for all site-directed mutagenesis according to the manufacturers’ instructions. Sanger DNA sequencing (Source Bioscience, Nottingham, UK) was used to confirm the mutated nucleotides and the deleted sequences.

### Homologous recombination repair assay

DSB repair efficiency was measured as described previously^16^. Stable MRE11 knockdown cells were generated from U2OS-I-SceI-GFP using psilencer-2.1-U6 neo (#5764; Thermo Fisher, Scientific, Waltham, MA), as per manufacturer’s recommendations. The sequence of shMRE11 is mentioned in the ‘Transfection’ section of the ‘Material and methods’. In brief, 2.5 × 10^5^ cells were seeded onto 6-well plates and transfected with 2.5 μg of pcDNA3.3-FL-MRE11 or pcDNA3.3-TR-MRE11 using lipofectamine 3000, respectively. Following 24 h of transfection, the cells had 2.5 μg of I-SceI expressing plasmid added. The cells were allowed to grow for 24 h, followed by the measurement of GFP+ cells by flow cytometry, performed on a on a BD FACS DIVA instrument (BD Biosciences, Franklin Lakes, NJ). The data were analysed by FlowJo V10. One-way ANOVA was used to determine the statistical significance of the experiments.

### Western blotting

Protein lysis buffer comprised 50 mmol/L HEPES, 100 mmol/L NaCl, 10 mmol/L EDTA, 1% Triton X-100, 4 mmol/L Na pyrophosphate, 2 mmol/L sodium orthovanadate, 10 nmol/L NaF, and 50 mmol/L B-glycerophosphate. Cells were lysed in lysis buffer containing a cocktail of proteinase inhibitors (Roche, Mannheim, Germany). Protein quantification of the lysates was performed using a BCA protein assay (Thermo Fisher, Waltham, MA) and 30 µg of protein was resolved on 4–20% polyacrylamide gels and transferred onto nitrocellulose membranes. The resulting membranes were incubated with blocking buffer (Li-cor Biosciences, Lincoln, NE) and primary antibodies. The antibodies used were anti-MRE11 (ab214; abcam, Cambridge, UK), anti-MRE11 C-terminal (ab227452; abcam, Cambridge, UK), anti-MRE11 N-terminal (sc-135992; Santa Cruz, Dallas, TX), anti-myc-tag (2276 S; CST, Danvers, MA), anti-phospho-histone H2A.X (Ser139) (2577; CST, Danvers, MA), anti-SPRTN (HPA025073; Atlas antibodies, Bromma, Sweden), anti-cdc25A (3652; CST, Danvers, MA), anti-pAKT (S473) (4060; CST, Danvers, MA), anti-VCP(p97) (10736-1-AP; Proteintech, Rosemont, IL), and mouse monoclonal anti-β-actin (ab6276; abcam, Cambridge, UK). Fluorochrome-conjugated secondary antibodies (925-68021, 926-32210; Li-cor Biosciences, Lincoln, NE) were detected by infrared scanning densitometry using the Li-cor Odyssey Infrared Detection System (Li-cor Biosciences, Lincoln, NE).

### Immunoprecipitation

Cell lysates were prepared in Pierce IP lysis buffer (Thermo; 87788). Total protein concentration was determined by BCA assay and 500 μg lysate was incubated with anti-MRE11 (ab214; abcam, Cambridge, UK) at 4 °C overnight on a tube rotator and then added to protein A/G plus agarose (sc2003; Santa Cruz, Dallas, TX) for 1 h at 4 °C. The beads were washed five times in PBS-Tween 0.05% and binding proteins eluted by boiling (95 °C for 10 min) in 40 μL Laemmli buffer before SDS-PAGE gel running. Ten percent of the total lysate was retained as the load fraction. The antibodies used were anti-MRE11 (ab30725; abcam, Cambridge, UK), anti-RAD50 (3427; CST, Danvers, MA), anti-NBS1 (NB100-143; Novus Biological, Centennial, CO), anti-myc-tag (2278, CST, Danvers, MA). To isolate SPRTN-interacting proteins, whole cell lysates were prepared in lysis buffer (50 mM Tris-HCl pH7.4; 150 mM NaCl; 0.1% NP-40; 1 mM EDTA, and 5% glycerol; protease and phosphatase inhibitors) containing 500 U/ml of benzonase at 4 °C. Flag-tag protein complexes were separated using the anti-flag M2 antibody (F1804; Merck, Whitehouse Station, NJ) and washed five times in IP buffer containing 0.05% NP-40 and eluted in 3xFlag peptide (F4799; Merck) for 30 min at RT.

### Clonogenic assay

Established stable cell lines were plated in 6-cm culture dishes containing 4 ml of fresh medium with appropriate numbers in triplicate irradiated at 0–8 Gy on the day after cell plating and then incubated for 2 weeks. Cells were stained with crystal violet staining solution (0.5%) in 80 ml distilled water, 20 ml methanol and 0.5 g crystal violet powder (Merck, Whitehouse Station, NJ). Colonies containing more than 50 cells were counted and the surviving fraction was determined as the total number of colonies formed divided by the total number of cells plated multiplied by the plating efficiency, as determined in untreated cells.

### Cell cycle and apoptosis analysis

For cell cycle analysis, cells were seeded in 10-cm culture dishes at a density of 3 × 10^6^ cells/dish. After 24 h, two different doses of radiation (1 Gy, 5 Gy) were applied and cells were incubated for 16 h. Cells were rinsed with PBS, harvested with 0.05% trypsin, and fixed in 70% ethanol overnight. Cells were stained with 25 μg/ml propidium iodide (PI) in 3.3 μg/ml ribonuclease A and 0.25% triton X-100, and analysed on a FACS flow cytometer (BD FACSCalibur, BD Biosciences).

FITC Annexin V (cat no. 556547, BD Pharmingen, San Diego, CA) was used to quantitatively determine the percentage of cells undergoing apoptosis according to the manufacturer’s instruction. Established stable cell lines were plated in 10-cm culture dishes containing 10 ml of fresh medium in appropriate numbers, irradiated to 4 and 8 Gy the day after cell plating and then incubated for 48 h before harvest.

### Immunofluorescence

Established stable cell lines were plated onto 8-well chamber slides (734-2050; NUNC, Roskilde, Denmark) and fixed with 100% methanol for 5 min at −20 °C, at each time point after IR. Cells were then washed for 5 min three times in PBS. All subsequent steps were carried out at room temperature. Samples were blocked for 30 min in blocking buffer (1% BSA, 22.52 mg/ml glycine in PBST (PBS + 0.1% Tween 20)). Rabbit anti-phospho-histone H2A.X (Ser139) (#2577; CST, Danvers, MA) and mouse anti-MRE11 (ab214; abcam, Cambridge, UK) were diluted 1:800 and 1:500, respectively in PBST and incubated overnight at 4 °C. Cells were washed with PBS, after which secondary antibodies [AlexaFluor 568 (A11036) and AlexaFluor 488 (A21202; Thermo Fisher Scientific, Waltham, MA)] were diluted 1:2000 in PBST and applied for 1 h at room temperature. Cells were washed with PBS as before and mounted using proLong diamond antifade mountant with DAPI (P36962; Thermo Fisher Scientific, Waltham, MA). Cells were visualised for γH2AX immunostaining using confocal microscopy (Zeiss 710; Zeiss, Oberkochen, Germany).

### RNA interference and plasmid transfection

siRNA for SPRTN (5′-GUCAGGAAGUUCUGGUUAA-3′)^17^ transfections were performed using Lipofectamine RNAiMax reagent (Thermo Fisher Scientific, Waltham, MA) according to the manufacturer’s instructions. Depletion was assayed 72 h post-transfection. For SPRTN overexpression, both pcDNA3.1-flag-SPRTN WT and SPRTN E112A plasmids were used for transfection and this was performed using Lipofectamine 3000 reagent (Thermo Fisher Scientific, Waltham, MA) according to the manufacturer’s instructions. Overexpressed SPRTN levels were assayed 16 h post-transfection.

### In vitro cleavage of MRE11

Recombinant SPRTN wt or E112A mutant were purified from *E. coli* as detailed in ref. ^18^. Commercial MRE11A (NM_005590) human recombinant protein was purchased from Origene (Rockville, MD). Cleavage reactions were performed in a 25 µL volume containing 500 ng MRE11, 2 µg SPRTN, and a 100 bp dsDNA oligonucleotides (30:1 molar ratio SPRTN:dsDNA) in cleavage buffer (25 mM Tris, pH7.4; 150 mM NaCl) at 37 °C for overnight (>16 h). The reaction was stopped by the addition of Laemmli buffer and boiling and used immediately.

### Quantification and statistical analysis

Counting of γH2AX foci was determined using Image J software. For clonogenic assays, the surviving fraction was calculated on the basis of the number of colonies on non-irradiated plates. Data are presented as the log of the surviving fraction with error bars representing the SEM. Radiation survival curves were plotted in GraphPad Prism 8, using the linear-quadratic model with the equation SF = exp – (*αD* + *βD*^*2*^), where *D* is a dose of radiation. The FP values were plotted against the log of the protein concentrations, and the apparent dissociation constant calculated from fitting the curves with a sigmoidal four-parameter logistic binding model in GraphPad Prism 8. Spearman’s correlation value was acquired through ImageJ with colocalisation plugins, and P values for one-way ANOVA multiple comparison was calculated in GraphPad Prism 8. The levels and correlation between SPRTN and TR-MRE11 were calculated using a 2-way ANOVA multiple comparison and correlation, respectively, in GraphPad Prism 8.

## Results

### MRE11 in primary bladder tumours shows a truncated form which lacks the C-terminus

**Fig. 1:**
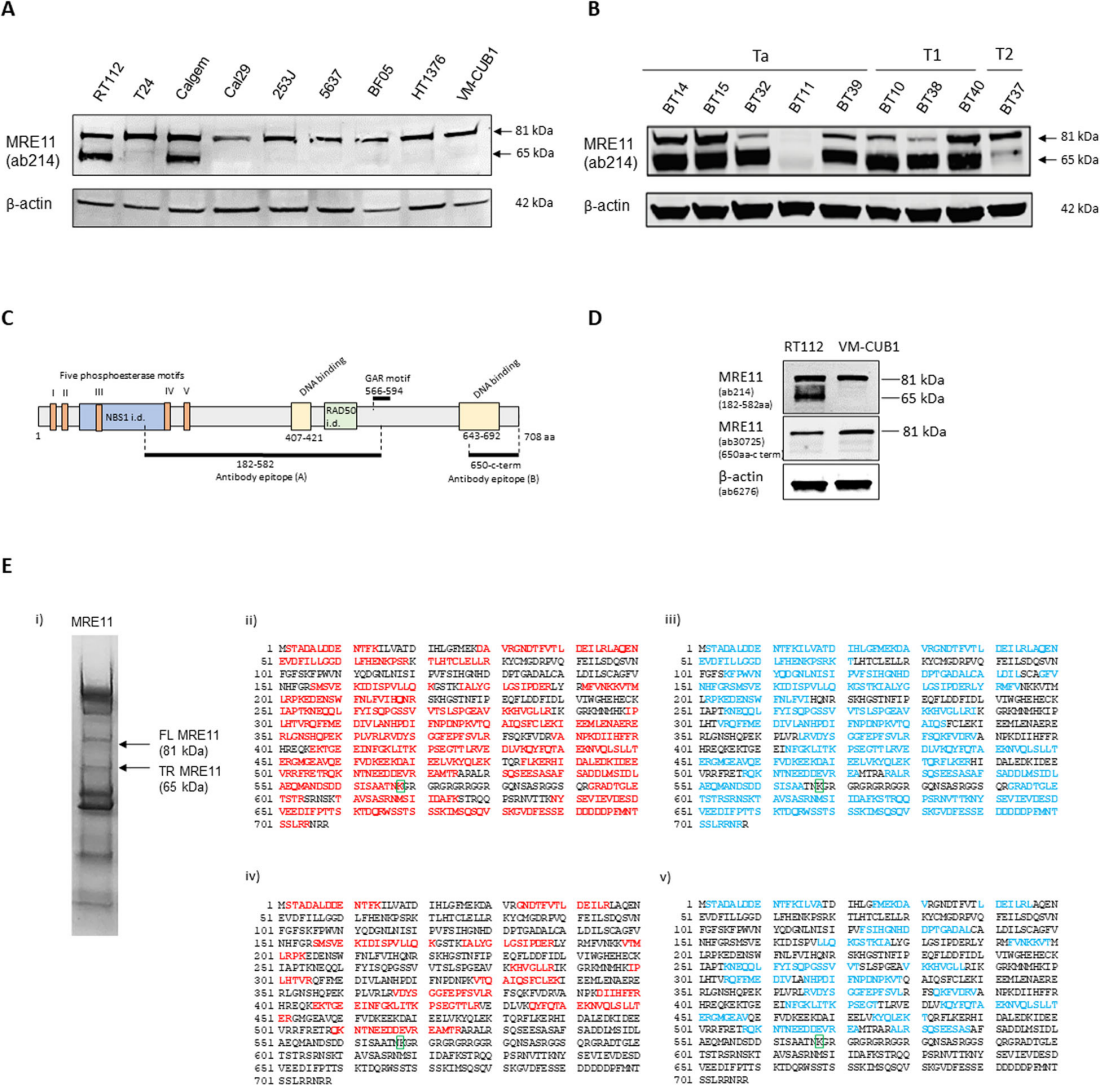
MRE11 is found in truncated form in bladder cancer cell lines and primary bladder tumours. **A** Two out of nine bladder cancer cell lines show a truncated form of MRE11 (approximately 65 kDa). Calgem is gemcitabine-resistant cells created from Cal29 by exposure to increasing gemcitabine doses^49^. **B** Eight out of nine nuclear cell-free extracts from human bladder tumours show a truncated form of MRE11 (approximately 65 kDa). Ta = papillary tumour, T1 = tumour invading lamina propria, T2 = tumour invading muscularis propria. **C** Schematic diagram of MRE11 and the epitope regions of MRE11 of two antibodies, spanning amino from acids 182 to 582 (ab214) and 650 to C-terminus (ab30725), respectively. **D** In RT112, ab214, spanning amino acids 182–582, detected both FL and TR MRE11, but not in VM-CUB1. In contrast, the C-terminus anti-MRE11 antibody (ab30725) did not detect the truncated MRE11 both in RT112 and VM-CUB1. **E**-i The eluted sample from super-paramagnetic beads with recombinant protein A-bound MRE11 was loaded onto a precast 4–20% polyacrylamide gel. **E**-ii, iii The sequence coverage of FL-MRE11 digested with trypsin (red) and elastase (blue) was 71% coverage for both cases. **E**-iv, v Protein sequence coverage of TR-MRE11 digested with trypsin (red) and elastase (blue) were 23% and 28% respectively. The green box at K568 indicates the last amino acid of TR-MRE11 model.

Having previously identified a C-terminally truncated form of MRE11 (TR-MRE11) in the bladder cancer cell line RT112, and in T24 bladder cancer cells following HDAC inhibition by panobinostat treatment^13^, we extended our cell line panel to include seven other bladder cancer cell lines (Fig. 1A). In cell-free extracts taken from primary bladder tumours at transurethral tumour resection, we also found the presence of the truncation in tumour samples from bladder cancer patients, but this was not associated with clinical stage (Ta, T1 and T2) (Fig. 1B).

We used two different antibodies against MRE11, which bind to the central (ab214, 182–582aa) or C-terminal regions (ab30725; 650aa-C-term) of full length MRE11, respectively, to further elucidate the truncated phenotype (Fig. 1C). TR-MRE11 was not detected with the antibody ab30725 which targets an epitope in the C-terminal domain in either RT112 or VM-CUB1 cell lines. However, both forms were detected by the antibody ab214, targeting amino acids 182–582 in RT112 but not in VM-CUB1 cells (Fig. 1D). This indicate that TR-MRE11 does not include the C-terminal region of MRE11, and not all cell lines express TR-MRE11 forms at baseline.

Previously, our group demonstrated the existence of TR-MRE11 by mass spectrometric analysis^13^. To identify the MRE11 truncation locus, we optimised the MRE11 pull down by increasing the initial amount of protein and repeated the mass spectrometry experiment using two different proteases (trypsin and elastase), in order to maximise protein sequence coverage (Fig. 1E). There was 71% protein sequence coverage following trypsin digestion and 71% coverage after elastase digest of the upper band representing FL-MRE11 (Fig. 1E-ii, iii). In the lower MW band, we detected peptides covering MRE11 without the C-terminus (31% and 38% coverage between 1 and 538 aa, respectively), with no coverage of sequence between 539 and 708 aa (Fig. 1E-iv, v). The confidence in protein identification, as represented by sequence coverage and emPAI, is shown in Table S1. We hypothesised that the putative cleavage site (PCS) of MRE11 would lie in the region between amino acid 539 and amino acid 600 for the following reasons: (1) Assuming that the tryptic digest was complete, the arginine located at amino acid 594 would have been cleaved by trypsin to generate a peptide of 8-20 amino acid chain length detectable by Mass Spectrometry, if TR-MRE11were included that region. We, therefore, concluded that the PCS is located before MRE11 amino acid 594. (2) If TR-MRE11 were longer than 600 aa, the molecular weight of TR-MRE11 would be bigger than 68.4 kDa. However, the approximate size of TR-MRE11 on western blots was smaller than 67 kDa. In case the cleaving enzyme worked in a more distal region, we extended the range of the PCS to amino acid 600, rather than restricting it to amino acid 594.

### The cleavage site of MRE11 is between amino acids 559 and 580

We established a stable MRE11 KD cell line using shMRE11 and found a 5.6-fold decrease in MRE11 expression compared to WT (Fig. S1A–C).

**Fig. 2:**
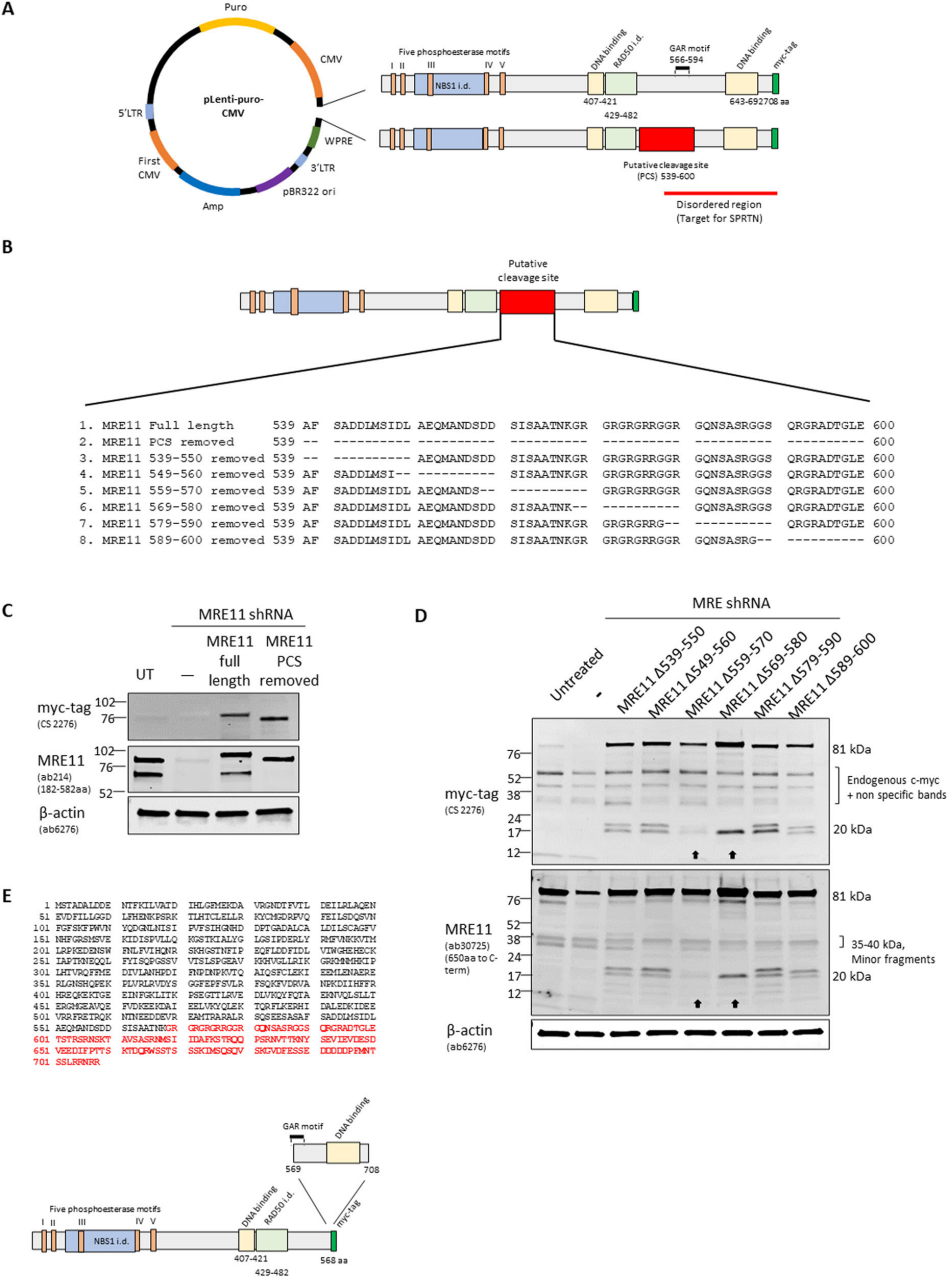
The putative cleavage site in MRE11 lies within a 22 amino acid region, namely amino acids 559–580. **A** A map of the lentiviral vector including either intact MRE11 or MRE11 with the initial putative cleavage site (PCS) at amino acids 539–600 removed. The functional domains of MRE11 are indicated^50,51^. **B** Schematic representation of MRE11 with the various mutants, including complete deletion of the region (MRE11 PCS removed) and sequential deletions of 12 amino acids (MRE11 Δ539–550, Δ549–560, Δ559–570, Δ569–580, Δ579–590 and Δ589–600). **C** The MRE11 mutant lacking the initial putative cleavage site failed to generate truncated MRE11. **D** MRE11 mutants lacking either 559–570 or 569–580 aa did not generate the approximately 20 kDa size short form of cleaved MRE11 in RT112 cells, as seen using anti-myc-tag and anti-MRE11 ab 30725 antibodies. **E** pLenti-TR-MRE11 vector was designed by deleting 569–708 aa from FL-MRE11 (amino acids deleted shown in upper panel). Lower panel shows the truncation in diagrammatic form.

We removed the region of the PCS, namely, amino acids 539–600 (Fig. 2A) from the MRE11 plasmid. MRE11-wt and PCS deleted (MRE11 Δ539–600) plasmids were independently transfected into RT112 MRE11 KD cells, and transfection efficiency determined by western blot against the C-terminal myc-tag (Fig. 2C, S2). The cleaved C-terminal part of MRE11 was detected in FL-MRE11 expressing cells but was absent in cells lacking the PCS (MRE11 Δ539–600 aa), implying that MRE11 cannot be cleaved when amino acids 539–600 are absent (Fig. 2A). In order to further specify the PCS, we designed a set of plasmids expressing various forms of MRE11 mutants, to establish stable cell lines, using a myc-tag on the C-terminal region of MRE11 (Fig. 2B). Thirty-eight cell lines from six different deletions between 539 and 600 aa were generated and representative cell lines with similar myc-tag expression were selected (Fig. S2). The six deletions were: 539–550 aa, 549–560–aa, 559–570 aa, 569–580–aa, 579–590 aa and 589–600 aa. The presence of two small fragments containing myc-tag (~20 kDa) suggested that MRE11 has two cleavage sites very near each other. The higher of the two bands (~20 kDa) was absent in the Δ559–570 aa and Δ569–580 aa deletion mutants, implying that the cleavage site is located between amino acids 559 and 580 aa (Fig. 2D). Thus, we designated 1–568 aa as TR-MRE11 because from this experiment the cleavage site was determined to lie at 569 ± 11 aa and, furthermore, a run of glycine-arginine repeats started from amino acid 569. We, therefore, removed amino acids 569–708 to generate a close representation of the TR-MRE11 form in the lenti-viral vector (Fig. 2E). Of note, the level of intrinsic disorder is very high after 500 aa in MRE11, according to the predicted secondary structure of MRE11 (Fig. S3), and this could be one of the reasons why FL-MRE11 is not stable at a post-translational level, allowing TR-MRE11 forms to appear.

### TR-MRE11 forms the intact MRN complex but lacks nuclease activity

**Fig. 3:**
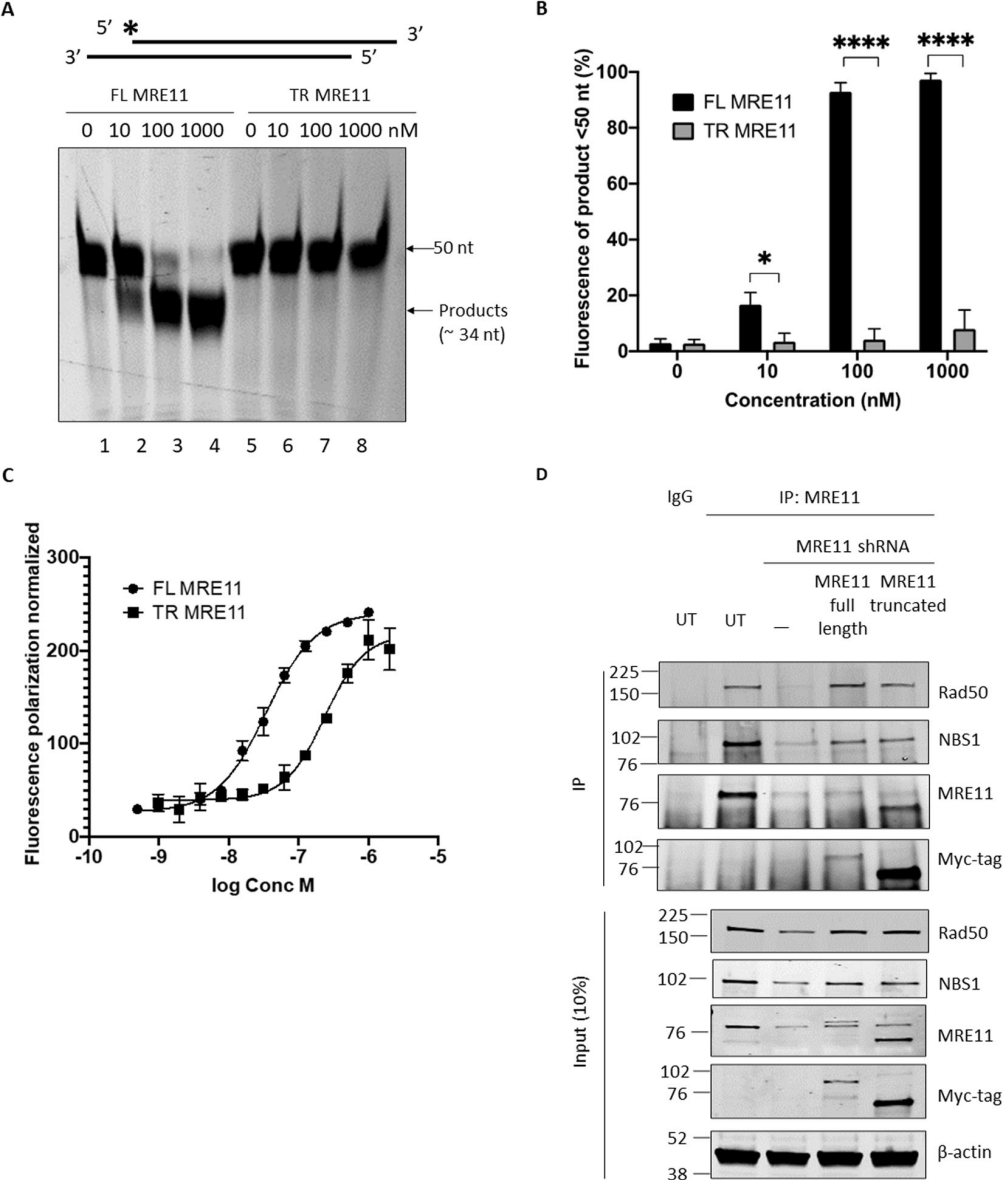
TR-MRE11 lacks nuclease and DNA binding activities. **A** Nuclease assays of FL-MRE11 (lane 1, 2, 3, 4) and TR-MRE11 (lane 5, 6, 7, 8) on 3′-overhang DNA substrates (*n* = 3). The FL-MRE11 shows clear dose-dependent nuclease activity which is absent in TR-MRE11. **B** Quantification of nuclease activity data in (**A**). Error bars represent SEM (*P* < 0.0001 (****), *P* < 0.05 (*)). **C** Measurement of fluorescence polarisation (*n* = 1). The apparent dissociation constant for FL-MRE11 was 34.56 nM, whilst TR-MRE11 showed significantly reduced DNA binding (242.15 nM). **D** TR-MRE11 was still able to efficiently interact with RAD50 and NBS1, and this has been shown through immunoprecipitated MRE11 from VM-CUB1 protein lysates.

Its dual 3′−5′ exo- and endonuclease activity is one of the most important biochemical features of MRE11^19^. To determine whether the C-terminal truncation of MRE11 affected the enzymatic activity of MRE11, purified FL-MRE11 (MRE11 wild-type), and TR-MRE11 (1–568 aa only) recombinant proteins (Fig. S4A, S4C) were identified and analysed by ESI-MS (Fig. S4B, S4D). When compared to FL-MRE11, the nuclease activity of TR-MRE11 on partial duplex dsDNA was abolished, in a range of concentrations between 10 and 1000 nM (1:10, 1:1, 10:1 molar ratio protein:dsDNA) (Fig. 3A, B). The nuclease products appeared to migrate approximately 34 nucleotides, consistent with the junction of the double stranded regions and what has been observed previously^20^. To determine whether defective DNA binding was responsible for the lack of nuclease activity, we performed a fluorescence polarization-based DNA binding assay^21^ sing the same substrates as in the nuclease assay (Fig. 3C). The apparent affinity of TR-MRE11 was 7-fold lower than FL-MRE11 (242.15 nM and 34.56 nM, respectively). These data demonstrate that the C-terminus contributed significantly to the DNA binding capacity and nuclease activity of MRE11.

The MRE11 dimer is the core scaffold, and the correct function of the MRN complex could be provided by the flexibility of its interaction with RAD50 and NBS1, which optimises MRN’s allosteric organisation^22–24^. We investigated TR-MRE11’s interaction with RAD50 and NBS1 by immunoprecipitation. Despite missing C-terminal motifs, TR-MRE11 efficiently interacted with RAD50, while NBS1 expression show a mild decrease over time (Fig. 3D, S6B). Altogether, these results suggest that TR-MRE11 maintains an intact MRN complex but the nuclease activity of TR-MRE11 is abolished due to deficient DNA binding.

### γH2AX foci co-localised with FL-MRE11 but not with TR-MRE11 after IR

**Fig. 4:**
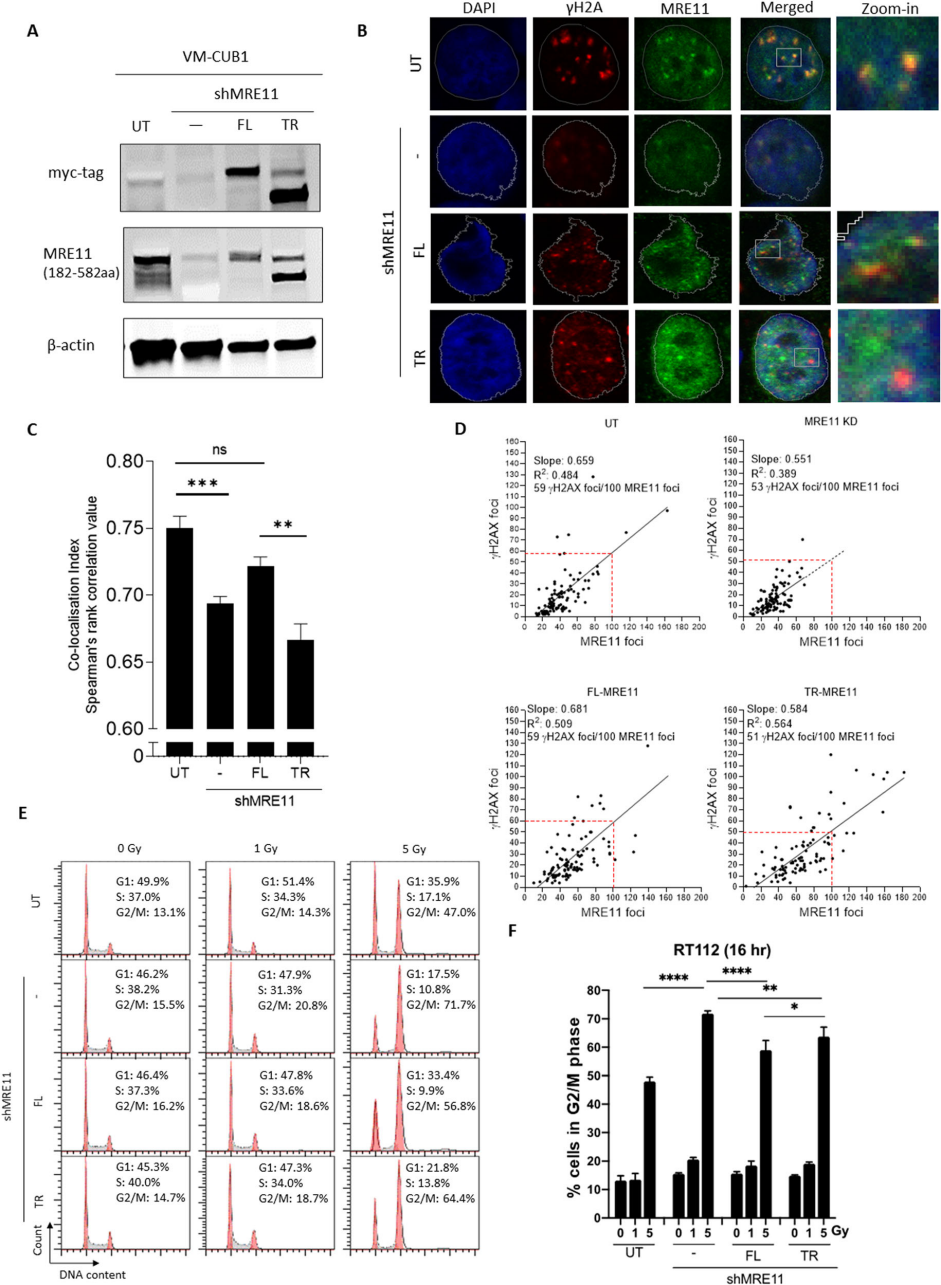
TR-MRE11 co-localises less frequently than FL-MRE11with γH2AX after IR in VM-CUB1 cells. **A** Western blot of established cell lines from VM-CUB1. Endogenous MRE11 was knocked down and replaced with either exogenous FL or TR-MRE11, and named MRE11 knockdown (KD), FL-MRE11, and TR-MRE11 cells, respectively. **B** Immunofluorescence microscopy of MRE11 and γH2AX foci showing co-localisation and some failure of co-localisation after IR (2 Gy, 24 hr post-IR). MRE11 foci were clearly knocked down in the shMRE11 sample and rescued in FL and TR. **C** TR-MRE11 colocalises with γH2AX less than FL-MRE11 does. Data are presented as means ± SEM (*P* < 0.001 (***), *P* < 0.01 (**)). **D** Correlation between MRE11 and γH2AX foci numbers from (**B**). MRE11 deficient and TR-MRE11 cells showed non-significantly lower slopes for γH2AX foci compared to FL-MRE11 and untreated cells. **E** Representative graphs for propidium iodide cell cycle. **F** Cell cycle distribution of RT112 UT, KD, FL-MRE11, and TR-MRE11 cells was quantitatively analysed using flow cytometry (****<0.0001, **<0.01, *P < 0.05; one-way ANOVA, *n* = 3, mean + SEM).

We studied TR-MRE11 functions in bladder cancer cell lines of the two main molecular subtypes of muscle-invasive bladder cancer (RT112 for luminal and VM-CUB1 for basal). Monoclonal cell lines expressing FL or TR-MRE11 were established in both MRE11 KD RT112 and MRE11 KD VM-CUB1 bladder cancer cells using lentivirus (Fig. 4A, S5A-B).

While the MRN complex binds to DNA double-strand breaks (DSB)^25^, γH2AX is necessary for the recruitment of other factors to the sites of DNA damage^26^. MRE11 undergoes relocalisation in the nuclei during DNA replication in damaged cells and this relocalisation reflects the association of the complex with DNA damage^27^. We found that MRE11 colocalised with γH2AX at 24 h post-IR in WT and FL-MRE11, but colocalization levels between MRE11 and γH2AX foci were decreased in TR-MRE11 (Fig. 4B, C, S5C, D) in both cell lines.

In terms of γH2AX foci formation, using immunofluorescence microscopy, we investigated γH2AX and MRE11 foci at a 30 min timepoint after IR which is believed to be the peak time for formation of γH2AX foci. As the number of MRE11 foci was reduced by MRE11 KD and this could be rescued by either FL-or TR-MRE11 (Fig S5F) from MRE11 KD, no significant change in the number of γH2AX foci was detected between FL- and TR-MRE11 cell lines at 30 min post-IR (Fig S5G, H). However, the smaller numbers of γH2AX foci per MRE11 foci were observed in TR-MRE11 compared to FL-MRE11 in RT112 cell line (52 and 67 γH2AX foci per 100 MRE11 foci, respectively), consistent with the result of VM-CUB1 cell line (51 and 59 γH2AX foci per 100 MRE11 foci, respectively) after IR, which indicates that the presence of the MRE11 truncation alters the cell’s ability to increase the phosphorylation of H2AX in response to ionising radiation (post-IR, 24 hr) (Figs. S5C, E, 4B, D). Altogether, this suggests that TR-MRE11 is less able to colocalise with γH2AX, but that the truncation of MRE11 does not influence the number of γH2AX foci formed immediately after IR.

### TR-MRE11 cells have less efficient HR repair, and are more radiosensitive, with higher G2/M arrest than FL-MRE11 cells

**Fig. 5:**
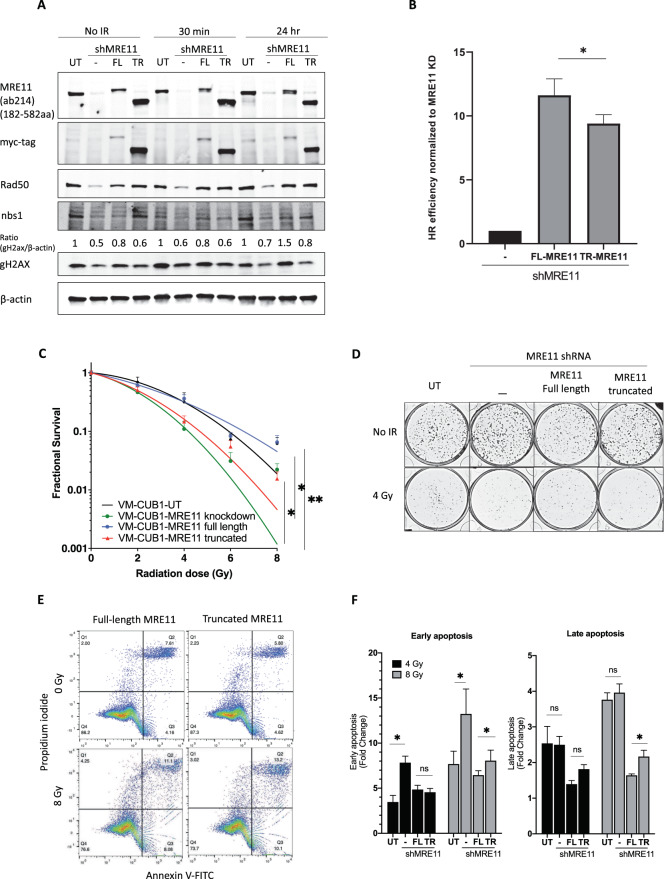
Cells expressing TR-MRE11 are more radiosensitive and deficiency in HR repair than those expressing FL-MRE11. **A** The protein expression levels of the MRN complex subunits were unchanged after IR (2 Gy). MRE11 KD and TR-MRE11 showed less increased level of γH2AX compared to WT after IR. **B** GFP based reporter assay showing the efficiency of HR repair pathway under FL-MRE11 or TR-MRE11 rescued condition as indicated. Graph represents the quantifications from three independent experiments (*P < 0.05; one-way ANOVA, *n* = 3, mean + SEM). **C** Clonogenic survival rates of TR-MRE11 and KD cells were lower than for FL-MRE11 and untreated cells. The response to ionising irradiation is represented by the linear-quadratic model. KD cells had significantly lower survival rate than UT cells (*P* < 0.05). TR-MRE11 cells had significantly lower survival rate than FL-MRE11 (*P* < 0.05) at 4 Gy. **D** The representative image of (**C**). **E** Flow cytometric analysis results of various RT112 cells after 8 Gy IR (48 h). **F** A statistical plot of annexin V-FITC/PI staining for (**E**) is shown for both early and late apoptosis after 4 and 8 Gy IR (48 h). The results are expressed as the means ± SD (****<0.0001, **<0.01, *P < 0.05).

The pattern of protein expression of the MRN components was consistent after IR, indicating that the overall protein level of each component of the MRN complex was unchanged following ionising radiation (Fig. 5A, S6A). MRE11 KD and TR-MRE11 cells had lower levels of γH2AX protein expression in both non-irradiated and irradiated samples compared to WT and FL-MRE11 samples, consistent with immunofluorescence results (Fig. 4D, S5D). The effect of the various MRE11 variants on the cell cycle was evaluated by flow cytometric analysis. We observed that MRE11 KD cells induced increased G2/M cell cycle arrest (4.64-fold) after radiation (5 Gy, 16 h), while control cells showed 3.65 times increase under the same conditions (*p* < 0.0001) (Fig. 4F). This result is also consistent with the recent report^28^ indicating that MRE11 deficit induces increased G2/M arrest. We additionally identified that TR-MRE11 rescued cells showed more G2/M arrest (4.31-fold) than FL-MRE11 rescued cells (3.78-fold) after IR (*p* < 0.05) (Fig. 4E, F), suggesting that TR-MRE11 might have inefficient HR causing cell cycle arrest.

To determine the role of the MRE11 C-terminus in the HR repair pathway, we used the DR-GFP reporter system in U2OS cells. We found that the FL-MRE11 and TR-MRE11 exhibited a significant increase in the restoration of the GFP+ cells over MRE11 KD cells (Fig. 5B). However, TR-MRE11 has been shown to significantly reduced HR repair efficiency when compared with the FL-MRE11, indicating the importance of C-terminal MRE11 in HR repair. Radiotherapy increase overall survival in the basal rather than the luminal cancer subtype^29^. Consistent with this, VM-CUB1 cells were more sensitive to radiation than RT112 cells in clonogenic assays (Fig. 5C, S6C). In both cell lines, the degree of radiation sensitivity in FL-MRE11 was similar to that of wild-type, while the degree of radiation sensitivity in TR-MRE11 was similar to that of MRE11 KD which is more radiosensitive (Fig. 5C, D, S6D). To further evaluate whether the MRE11 deficient cells’ sensitivity to IR is inducing apoptosis, we performed FACS analysis using FITC annexin V and PI (Fig. 5E, F). After both low (4 Gy) and high (8 Gy) doses of radiation, MRE11 KD showed significantly increased early apoptosis cells. TR-MRE11 cells had more early apoptosis cells than FL-MRE11 at the higher radiation dose, but not at the lower dose and not when compared to UT. No significant difference was observed in late apoptosis cells upon knockdown of MRE11 at either dose, although unexpectedly there was a difference between UT and transfected FL-and TR-MRE11 cells, both showing significantly less late apoptosis with this effect being more pronounced with the FL-MRE11. The differences in apoptosis do not fully explain the results in the survival assay, where a significant difference was observed between FL-and TR-MRE11 cells even at lower doses. MRE11 does appear to play some role in early apoptosis but this role is not particularly dependent on the C-terminus.

Taken together, these results indicate that the C-terminus, including the glycine-arginine-rich (GAR) domain, is important for choosing the more efficient DNA damage repair pathway, G2/M progression, and cell survival after IR, but does not appear to play a significant role in apoptosis.

### Formation of TR-MRE11 is not caspase-dependent

Previously, we showed that MRE11 cleavage occurs as a post-translational modification but that TR-MRE11 is not generated due to alternative splicing, because no alternative MRE11 transcripts were detected in amplified cDNA from MRE11^13^. To determine how the levels of FL or TR-MRE11 expression are regulated, we first verified which cellular processes contribute to the cleavage of FL-MRE11 to produce TR-MRE11. To investigate how TR-MRE11 is produced, we exposed cells to various forms of damage, including overgrowth, starvation, blockage of protein synthesis, induction of hypoxia, blocking of DNA synthesis, HDAC inhibition, ionising radiation, and AKT inhibition, as it is known the MRE11-dependent pathway can contribute to localisation of pAKT-S473^30,31^. Among these challenges only overgrowth significantly (*P* < 0.05) increased TR-MRE11 (Figs. S1, S7A). We found that TR-MRE11 expression depends on cell density. There was a 2.3-fold increase in TR-MRE11 with higher cell numbers (Fig. S1A–C). TR-MRE11 also increased in a cell density-dependent manner (Fig. S1D, E). In order to elucidate the mechanism, we first focused on the seven aspartic acids which might be a potential target for caspases. We tested a range of concentrations of the pan-caspase inhibitor Z-VAD-FMK (carbobenzoxy-valyl-alanyl-aspartyl-[O-methyl]-fluoromethylketone) in DMSO. MRE11 cleavage was not affected by inhibition with a range of doses of Z-VAD-FMK (Fig. S7B). We next tested individual caspase inhibitors 1, 2, 3, 4, 5, 6, 8, 9, 10 and 13, and again could not detect any significant alteration in TR-MRE11 expression levels (Fig. S7C). This suggests that caspases do not appear to be the proteases responsible for cleaving MRE11.

### MRE11 is a substrate of the SPRTN protease

We then chose to study the DNA-dependent mammalian metalloprotease, SPRTN, which is essential for DNA-protein crosslink (DPC) repair and DNA replication in vertebrate cells^32^. SPRTN is a DNA-dependent, but amino-acid sequence non-specific, protease. Its known substrates (e.g. histones) are cleaved in disordered protein regions in the vicinity of arginine, lysine and serine residues^18^. Thus SPRTN was a promising candidate responsible for the MRE11 cleavage, as the C-terminus of MRE11 is predicted to be intrinsically highly disordered (Fig. S3). Furthermore, the MRE11 GAR domain (Fig. 2A), located between 566–594 aa^33^, contains nine positively-charged arginines out of its 29 amino acids and is also disordered. This region could therefore be a possible candidate substrate for SPRTN.

**Fig. 6:**
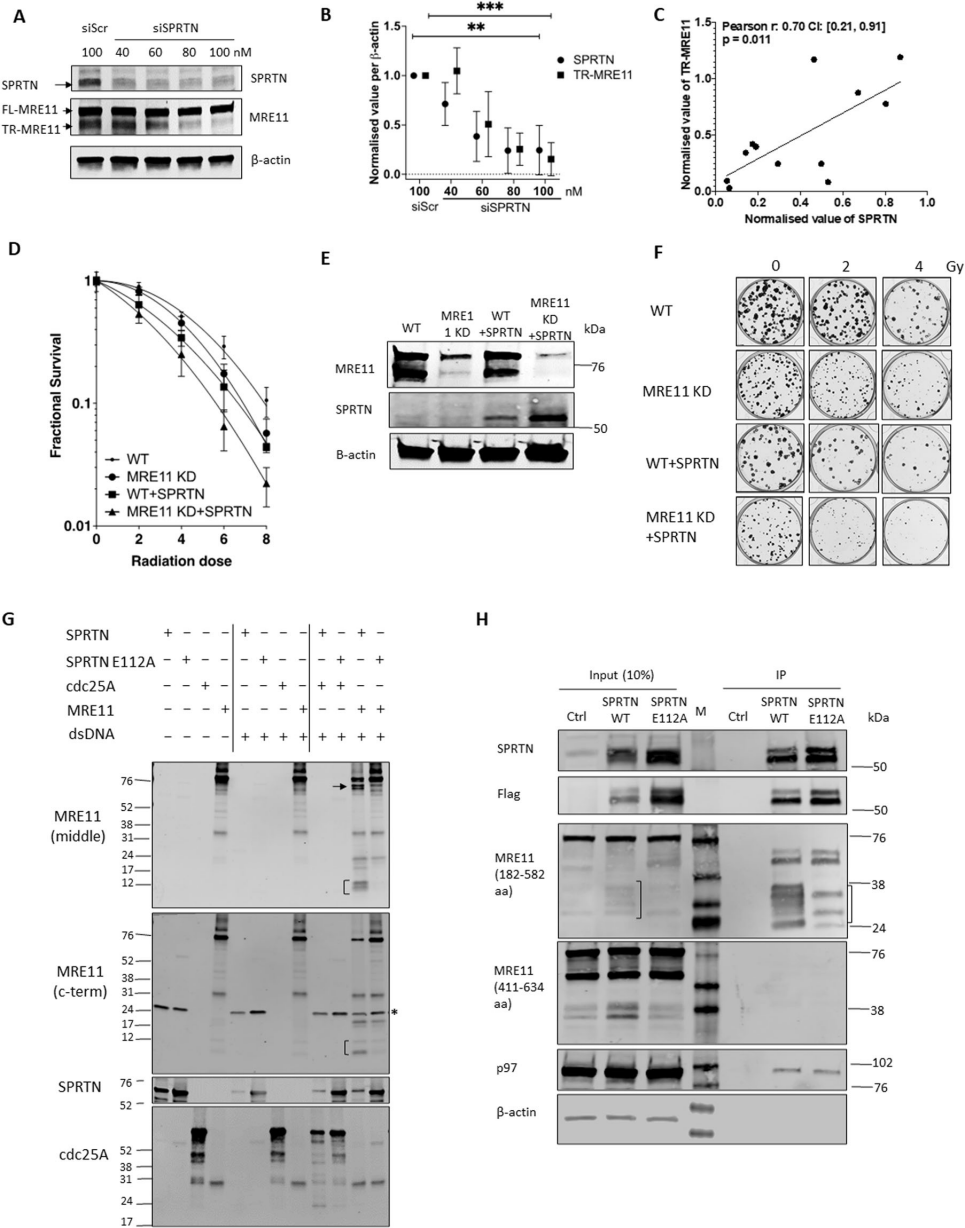
MRE11 is cleaved in cells and in vitro by the SPRTN metalloprotease and SPRTN overexpression increases radiosensitivity. **A** siRNA-targeting SPRTN in RT112 cells regulates the levels of TR-MRE11. siRNA was used indicated concentrations 40, 60, 80 and 100 nM for 72 h, with scrambled siRNA as a positive control. Pictures are representative from 3 replicates with similar results. **B** Quantification of TR-MRE11 and SPRTN knockdown level from (**A**). **C** A significant correlation was found between TR-MRE11 and SPRTN protein expression levels (*p* = 0.011). **D** Clonogenic survival rates of SPRTN overexpressed cells were lower after IR than for WT cells in RT112. The response to ionising irradiation is represented by the linear-quadratic model. MRE11 KD+SPRTN cells had significantly lower survival rate than WT+SPRTN cells (*P* < 0.05). WT+SPRTN cells had significantly lower survival rate than WT (*P* < 0.05). **E** SPRTN overexpression in WT and MRE11 KD cells was confirmed by Western blotting. **F** The representative images of (**D**). **G** In vitro cleavage assay using recombinant proteins. TR-MRE11, indicated with arrow, was released only in the presence of active SPRTN. A blot for anti-MRE11 (C-terminus) antibody shows nonspecific binding to SPRTN protein, marked with asterisk. Cdc25A was used as a positive control for SPRTN activity (the size of the cleaved products is ~5 kDa smaller than the products in Fig. 2D, as this recombinant protein does not include tag part in C-terminus). **H** Immunoprecipitation of 293T cell lysate using SPRTN-WT and SPRTN-E112A. p97, the strong interacting partner for SPRTN, was used as a positive control for SPRTN interacting protein.

To investigate whether post-translational MRE11 cleavage is mediated by SPRTN, endogenous SPRTN was depleted using siRNA targeting the 3′UTR of SPRTN transcripts. Knockdown of SPRTN resulted in a dose-dependent reduction in TR-MRE11 compared with scrambled siRNA-treated samples (Fig. 6A, B) although SPRTN knockdown did not influence to the protein expression level of RAD50 and NBS1, the MRE11’s interacting partners (Fig. S8C). There was a significant correlation between TR-MRE11 and SPRTN levels (Fig. 6C). Unexpectedly, SPRTN expression decreased upon treatment with HDACi (Fig S8A), and no effect was observed upon overgrowth (Fig S8B). We also observed large decrease in NBS1 and RAD50 upon treatment with panobinostat, suggesting that this affects levels of multiple cellular proteins. We overexpressed SPRTN protein to investigate whether this influenced survival following ionising radiation. Interestingly, overexpressed SPRTN increased sensitivity to radiation therapy, and this may be explained by the effect of SPRTN on MRE11 (Figs. 6D–F). Moreover, the increased IR sensitivity of SPRTN in MRE11 KD cells indicates that possible additional targets of SPRTN may be involved. Together, these results indicate that SPRTN is a factor mediating cleavage of MRE11 and overexpressed SPRTN increases radiosensitivity.

### SPRTN protease cleaves MRE11 in vitro

To further investigate the role of SPRTN protease in MRE11 cleavage, we performed an in vitro cleavage assay using recombinant proteins. Recombinant MRE11 protein was cleaved upon incubation with purified SPRTN recombinant protein and 100 bp dsDNA in vitro, but not with enzymatically-dead SPRTN variant *(SPRTN E112A)* (Fig. 6G). Incubation of MRE11 with SPRTN yielded a main N-terminal cleavage product of approximately ~65 kDa (arrow indicated) and others <17 kDa (bracket indicated), which were absent when SPRTN-E112A was used instead. In addition, the C-terminal binding antibody detected a small fragment (<17 kDa) specifically produced by SPRTN activity, reminiscent of the two small bands from the cell lysate at ~12 kDa (Fig. 2D). This demonstrates that the cleavage product generated by SPRTN is missing the C-terminal of MRE11, similar to the TR-MRE11 form in some bladder cancer cells (Fig. 1A, B). These results suggest that TR-MRE11 could be a SPRTN cleavage product. To investigate whether overexpressed SPRTN and MRE11 form a stable interaction, we performed immunoprecipitation using flag-SPRTN transfection. Intact SPRTN complex was pulled down with its interacting partners, and this was shown via anti-p97, one of the strongest interacting partners to SPRTN (Fig. 6H). We did not detect a direct interaction between SPRTN and FL-MRE11. Although the presence of a stable interaction is not necessary for the protease cleavage which can happen in a transient manner, we did detect some low molecular weight bands with the monoclonal anti-MRE11 targeting 182–582 aa epitope (Fig. 6H), which may correspond to MRE11 cleavage products that interact with SPRTN. Further studies would be required in order to investigate this possibility more thoroughly.

## Discussion

MRE11 has been intensively studied because of its importance in genome stability, DNA damage repair, and its nuclease activity. However, the reason why high MRE11 protein expression, by immunohistochemistry using an antibody with an epitope spanning amino acids 182–582, is associated with better survival rate in bladder cancer patients after radiotherapy^34,35^ has not yet been explained. We hypothesised that the truncated form of MRE11 may explain the negative correlation between the level of MRE11 expression and radiotherapy outcome, in terms of DNA repair.

Human MRE11 bears a glycine-arginine-rich (GAR) motif that is conserved among multicellular eukaryotic species^36^. They are essential for not only the regulation of MRE11 DNA binding and nuclease activity, but also MRE11 and RAD51 focus formation on a unique DSB in vivo^33^. Posttranslational modifications of MRE11, such as methylated arginine GAR motif and DSB processing, as well as ATR/CHK1 checkpoint signalling, appears to influence DNA damage repair and has been demonstrated previously^37^. According to Dery et al. 2008, MRE11-∆GAR on dsDNA displayed only weak residual exonuclease activity compared to that of methylated MRE11-WT. Additionally, we have shown in this current report that MRE11 recombinant protein without methylation would also have lack of nuclease activity after cleavage. Mutations in *MRE11* are responsible for the human radiation sensitivity disorder Ataxia-telangiectasia-like disorder (ATLD), which is characterised by defective checkpoint responses and high levels of chromosomal abnormalities^20^. The authors found that in ATLD 1/2 where MRE11 contained a stop codon instead of R633, ~50% residual nuclease activity remained. Both MRE11-∆GAR and ATLD 1/2 share a number of key features, such as having weak nuclease activity through loss of part of MRE11. Furthermore, this paper offers the evidence that the post-translationally induced TR-MRE11 forms (G569stop) lost its nuclease activity due to lack of DNA binding affinity (Fig. 3) reminiscent of MRE11-∆GAR and ATLD1/2. Despite this, TR-MRE11 interacted efficiently with RAD50 and NBS1 (Fig. 3D, S6B). As truncated MRE11 physiologically interacts with its MRN partners but is completely defective in nuclease activity, it is likely that the complex containing TR-MRE11 may have reduced DNA binding and nuclease activity, but this remains to be tested. Overall, a C-terminally truncated form of MRE11 is capable of maintaining the interactions between the MRN complex units while limiting its enzyme activity.

MRE11 participates in both NHEJ and HR DNA repair pathways^7,38^. Elimination of resection mechanisms (MRE11Δexo1Δsgs1Δ triple mutants) prevents HR and causes lethality in budding yeast^39^. Thus, we investigated whether FL-MRE11 and TR-MRE11 cells displayed similar ratios of HR repair pathway usage. In the HR assay used in this study, FL-MRE11 cells promoted efficient HR repair pathway compared to TR-MRE11 cells (Fig. 5B). Furthermore, Xu et al. showed that MRE11 knockdown caused radiosensitivity in an adenocarcinoma cell line^40^, which resembles our finding in this paper. C-terminally truncated MRE11 also caused radiosensitivity as MRE11 knockdown did but showed a lower degree of slope (Fig. 5C, D, S6C, D). The cell proliferation test using the MTT assay did not reveal any significant results after various doses of IR (Fig. S6E), indicating that MRE11 deficiency mediated cell survival and cell cycle progression but did not show dramatically altered cell proliferation rates.

Phosphorylated H2AX at ser 139 has been found at the sites of DSB in chromosomal DNA and γH2AX is a key component of various signalling pathways related to DNA DSB^41,42^. γH2AX focus formation is considered to be a sensitive signal for DNA DSB and necessary for rapid DDR signal amplification^43,44^. In addition, γH2AX directly binds NBS1 which then allows the recruitment of other DDR proteins, including the MRN complex at DNA DSB sites, and these foci co-localise with DNA repair and checkpoint proteins^26,45,46^. γH2AX recruits the MRE11 complex to damaged DNA over time^26,46^. The level of co-localisation with TR-MRE11 and γH2AX foci were significantly lower than that of FL-MRE11, indicating that the C-terminus of MRE11 is important for recognition of MRE11 recruitment by γH2AX (Fig. 4B, C, S5C, D).

Our findings also address another key question of what results in accumulation of TR-MRE11. The only factor that appeared to have an effect on TR-MRE11 levels was overgrowth or high cell density, which is known to affect multiple cellular processes (Fig. S7). Some possible caspase cleavage motifs including 560 (DSDD*SISA) aa in the PCS of MRE11 were identified by Cascleave^47^. However, we found no correlation (Fig. S7B, C) but found that SPRTN metalloprotease was involved. MRE11 cleavage was dependent on SPRTN and the level of TR-MRE11 was dependent on SPRTN expression (Fig. 6). These data indicates that SPRTN can induce MRE11 truncated forms in the cell. Further experiments are necessary to determine which of the domains in SPRTN is crucial for MRE11 cleavage, either through rescuing SPRTN-wt or SPRTN-mutated in SPRTN KD cells. Furthermore, the physiological importance of MRE11 cleavage in SPRTN-mediated DPC degradation should also be explored. The Cancer Genome Atlas (TCGA) data reveals that higher SPRTN levels were associated with decreased patient survival rates, and it was elevated in some of cancer types (Fig S8E, G). Furthermore, SPRTN levels were not significantly correlated with MRE11 levels and were independent of cancer stage (Fig S8D, F).

Previously, we found that bladder cancer patients with high MRE11 expression paradoxically have shown higher survival rates after radiation therapy, despite MRE11 being involved in repair of DNA DSB caused by ionising radiation^34^. This finding was validated by Laurberg et al. in an independent cohort. These findings might now be explained due to the presence of the truncated form of MRE11.

## Supplementary information

Supplementary data

## Data Availability

The LC-MS/MS proteomics data have been deposited to the ProteomeXchange Consortium via the PRIDE^48^ partner repository with the dataset identifier PXD017964 and 10.6019/PXD017964.
