# Supplementary material for: SPRTN protease-cleaved MRE11 decreases DNA repair and radiosensitises cancer cells
Source: Cell Death Dis. 2021 Feb 8;12(2):165. doi: 10.1038/s41419-021-03437-w (PMC7870818; doi:10.1038/s41419-021-03437-w)
Supplement: Supplementary file 1 — Supplementary data [file 41419_2021_3437_MOESM1_ESM.pdf]

Supplementary data

SPRTN protease-cleaved MRE11 decreases DNA repair and radiosensitises cancer cells

Juri Na[1], Joseph A Newman[2], Chee Kin Then[1], Junetha Syed[1], Iolanda Vendrell[1,3], Ignacio Torrecilla[1], Sophie Ellermann[1], Kristijan Ramadan[1], Roman Fischer[3], Anne E Kiltie[1]
[1]MRC Oxford Institute for Radiation Oncology, Department of Oncology, University of Oxford, Oxford, OX3 7DQ, UK
[2]Centre for Medicines Discovery, University of Oxford, United Kingdom
[3]Target Discovery Institute, Nuffield Department of Medicine, University of Oxford, Oxford, United Kingdom.

Corresponding author:
Prof Anne E Kiltie
MRC Oxford Institute for Radiation Oncology
Department of Oncology
University of Oxford
Oxford OX3 7DQ
anne.kiltie@oncology.ox.ac.uk

Conflicts of interest:
The authors have no conflicts of interest to declare.

Table S1 (related to Figure 1E)

			MASCOT output (20 ion cut off; 1% FDR)				
Protein Name	Protease (in-gel-digest)	Sample	score	sequence coverage	Num. of sequences	Num. of significant sequences	emPAI
Double-strand break repair protein MRE11	trypsin	TR MRE11	425	23%	17	10	0.52
		FL MRE11	2959	71%	58	41	12.49
Double-strand break repair protein MRE11	Elastase	TR MRE11	202	28%	35	9	0.46
		FL MRE11	1218	71%	134	46	9.38

Table S1 (related to Figure 1E). MASCOT protein identification parameters for MRE11 full length and truncated forms for both trypsin and elastase digestions. The table includes the mascot score (confidence on protein identification), sequence coverage, number of sequences and emPAI value, (exponentially modified protein abundance index; MASCOT Matrix Science). The formula for emPAI is: $emPAI = 10^{\frac{N_{observed}}{N_{obervable}}} - 1$. The high number of peptides, mascot scores and emPAI value corroborates the confident identification of MRE11 (truncated and full length) for both trypsin and elastase. The MASCOT protein identification parameters are calculated based on the full MRE11 sequence. For that reason, the sequence coverage, mascot scores and number of peptides for TR-MRE11 are lower due to the lack of C-terminus, nevertheless the values support a confident identification.

Figure S1 (related to Figure 1 and 6)

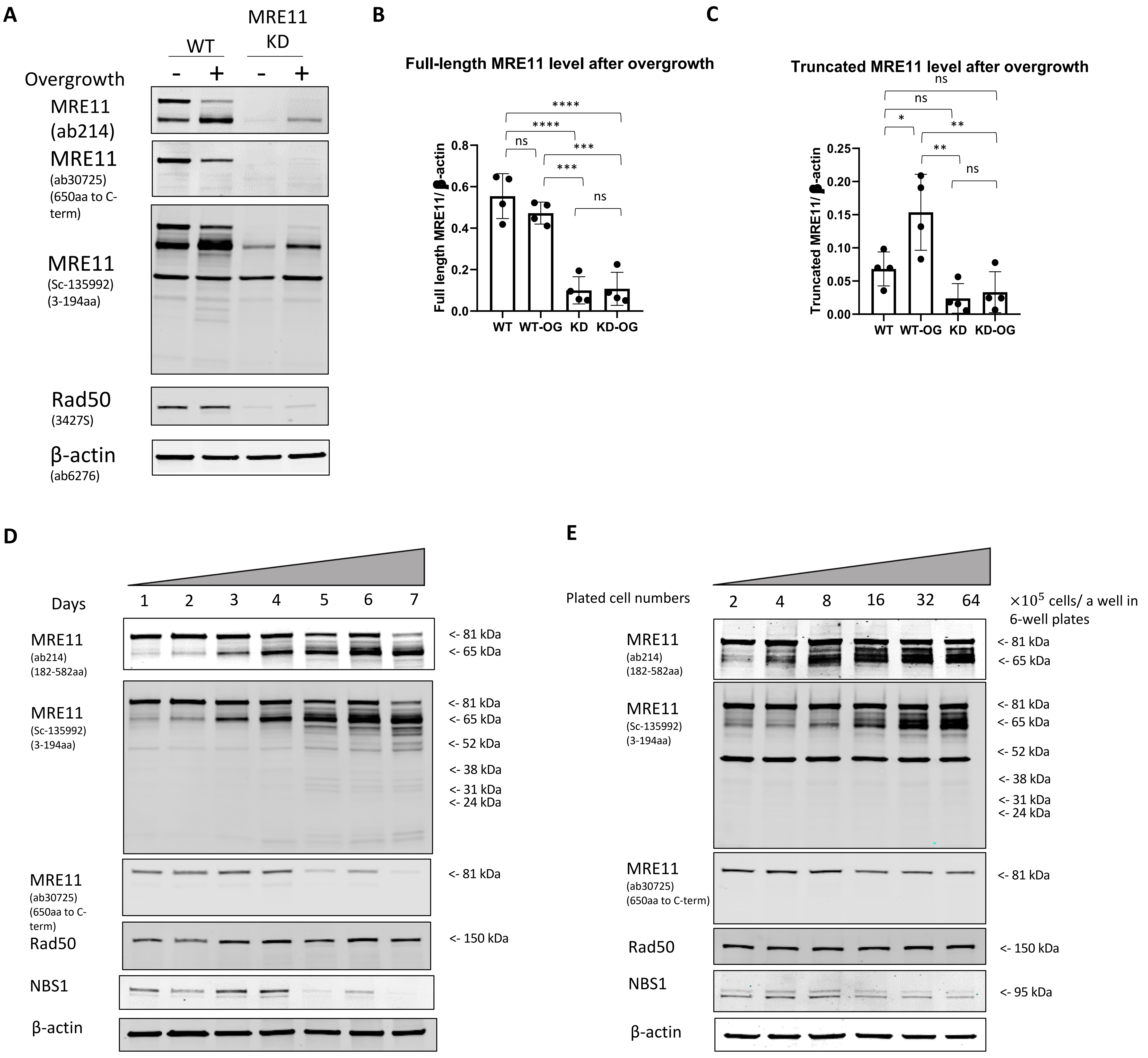

Figure S1 (related to Figures 1 and 6). The expression of TR-MRE11 was enhanced when cells were allowed to overgrow. (A) MRE11 was knocked down in the RT112 cell line, with a remarkable decrease in RAD50. Consistent results for MRE11 expression levels were formed for three different anti-MRE11 antibodies that bind to N-terminal (SC-135992), mid (ab214), c-terminus (ab20725). (B) Graph of FL-MRE11 levels showing dependence on cell density. (C) Graph of TR-MRE11 level showing dependency on cell density (P < 0.0001 (****), P < 0.001 (***), P < 0.01 (**), P < 0.05 (*); one-way ANOVA, n=3, mean +SEM). (D) Cells were harvested daily for 7 days. TR-MRE11 levels increased over time, with reduction in both FL-MRE11 and NBS1 from Day 5. The level of Rad50 was consistent across all time points. (E) Different numbers of cells were seeded and higher cell seeding numbers resulted in stronger TR-MRE11 expression.

Figure S2 (related to Figure 2)

A

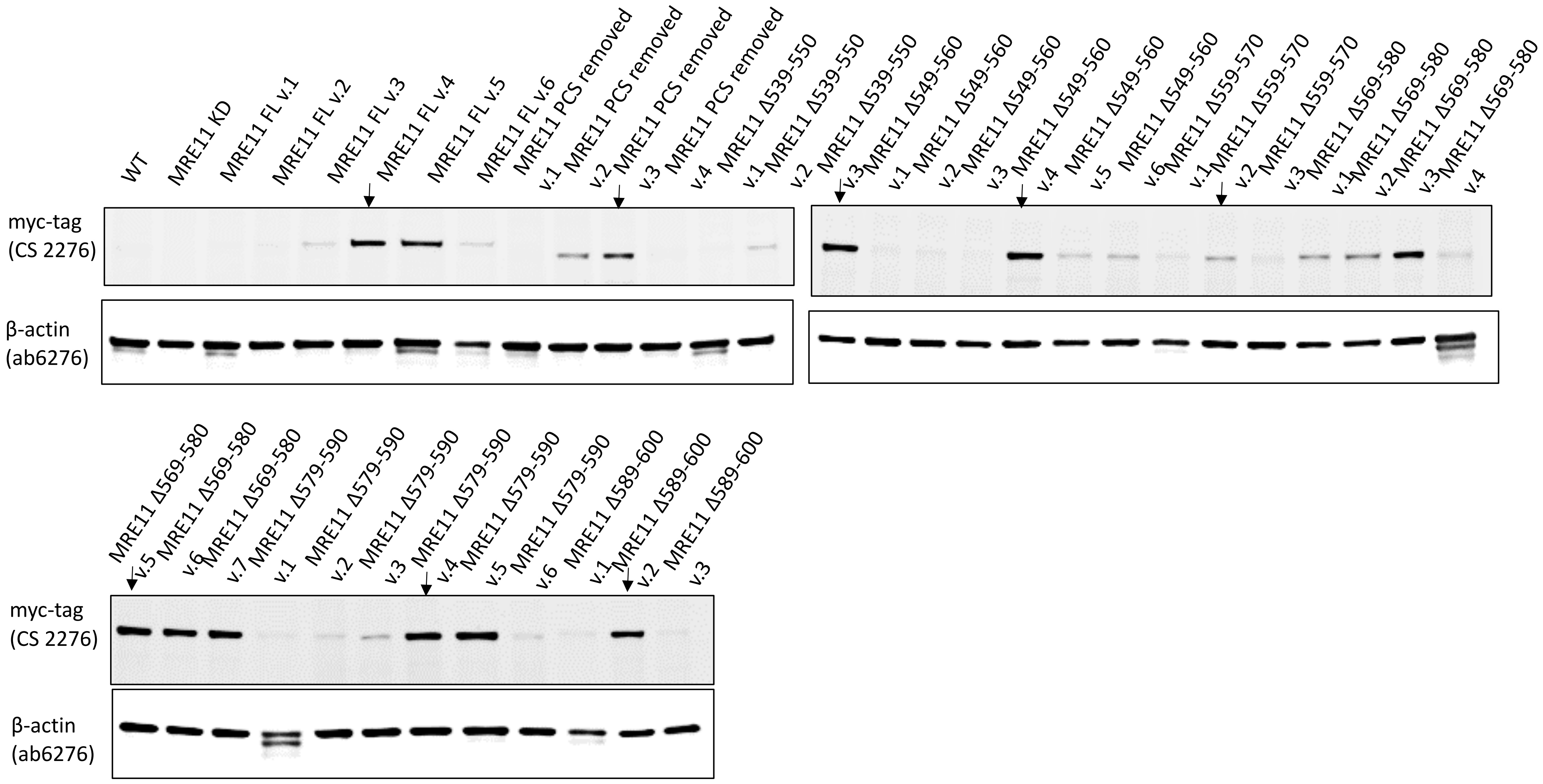

Figure S2 (related to Figure 2). Screening of transfection efficiency in myc-tagged samples. As the myc-tag is attached to the MRE11 c-terminus of MRE11 in the lenti-viral vector, transfection efficiency could be determined by myc-tag expression level. The following were selected as representative samples expressing similar levels of myc-tag: MRE11 PCS removed (Δ539-600 aa), Δ539-550 aa, Δ549-560 aa, Δ559-570 aa, Δ569-580 aa, Δ579-590 aa, and Δ589-600 aa.

Figure S3 (related to Figure 3)

A

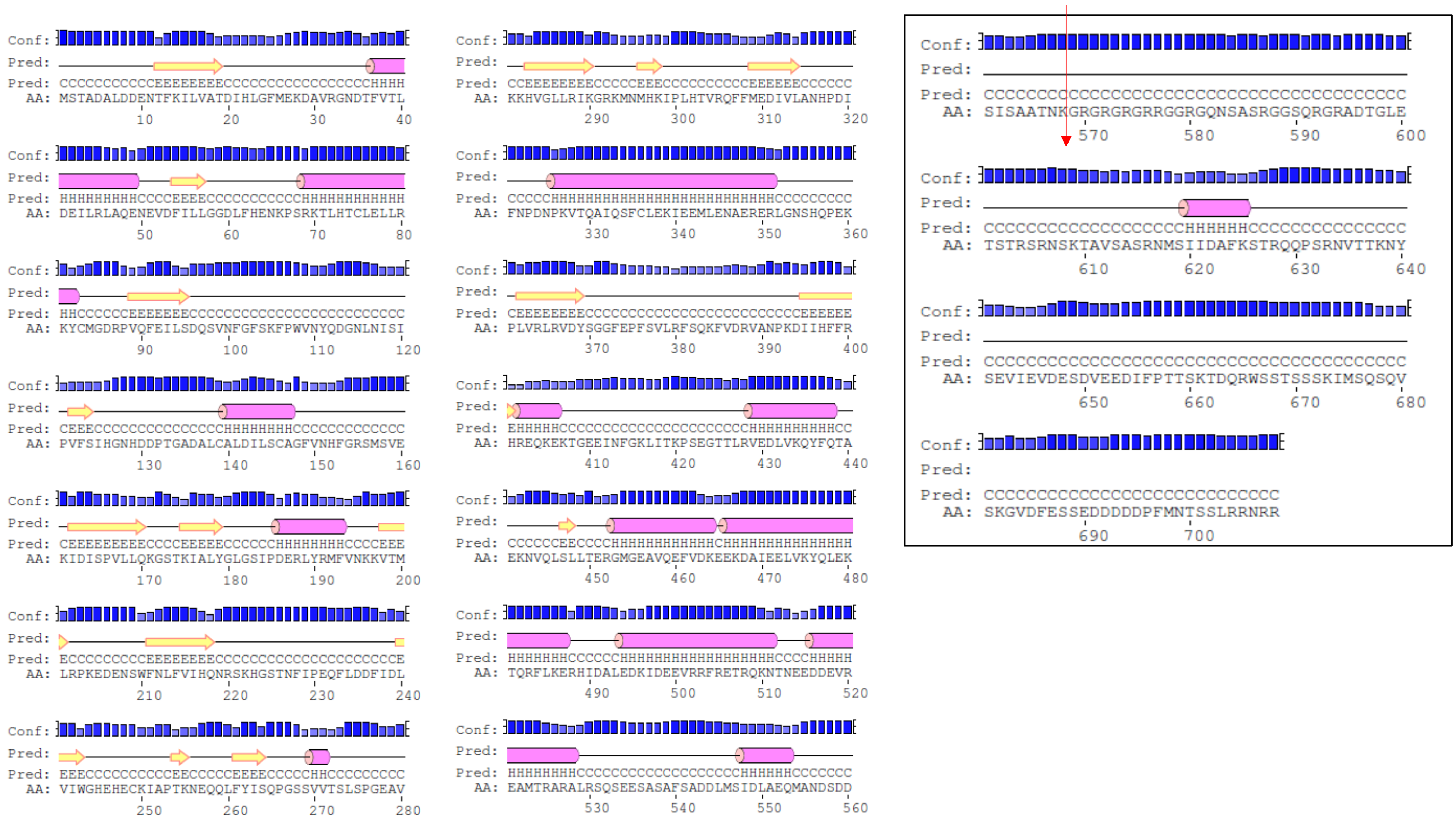

B

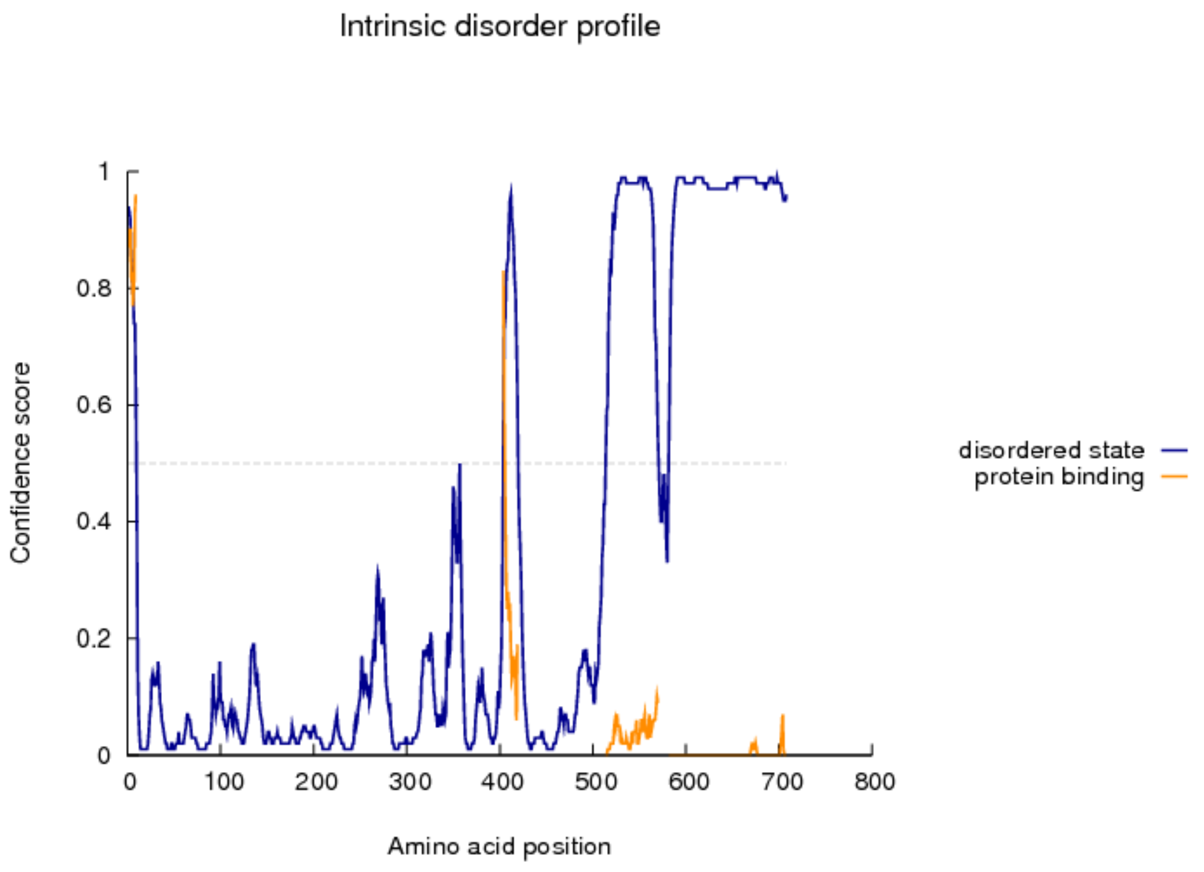

Figure S3 (related to Figure 3). The C-terminal part of MRE11 is very disordered. (A) PSI-Blast-based secondary structure PREDiction (PSIPRED, UCL, London) shows that the 569-708 aa region is intrinsically disordered, which means that the overall structure of TR-MRE11 is relatively ordered. (B) Graph showing the disordered state of MRE11 is very high after amino acid 500.

Figure S4 (related to Figure 3)

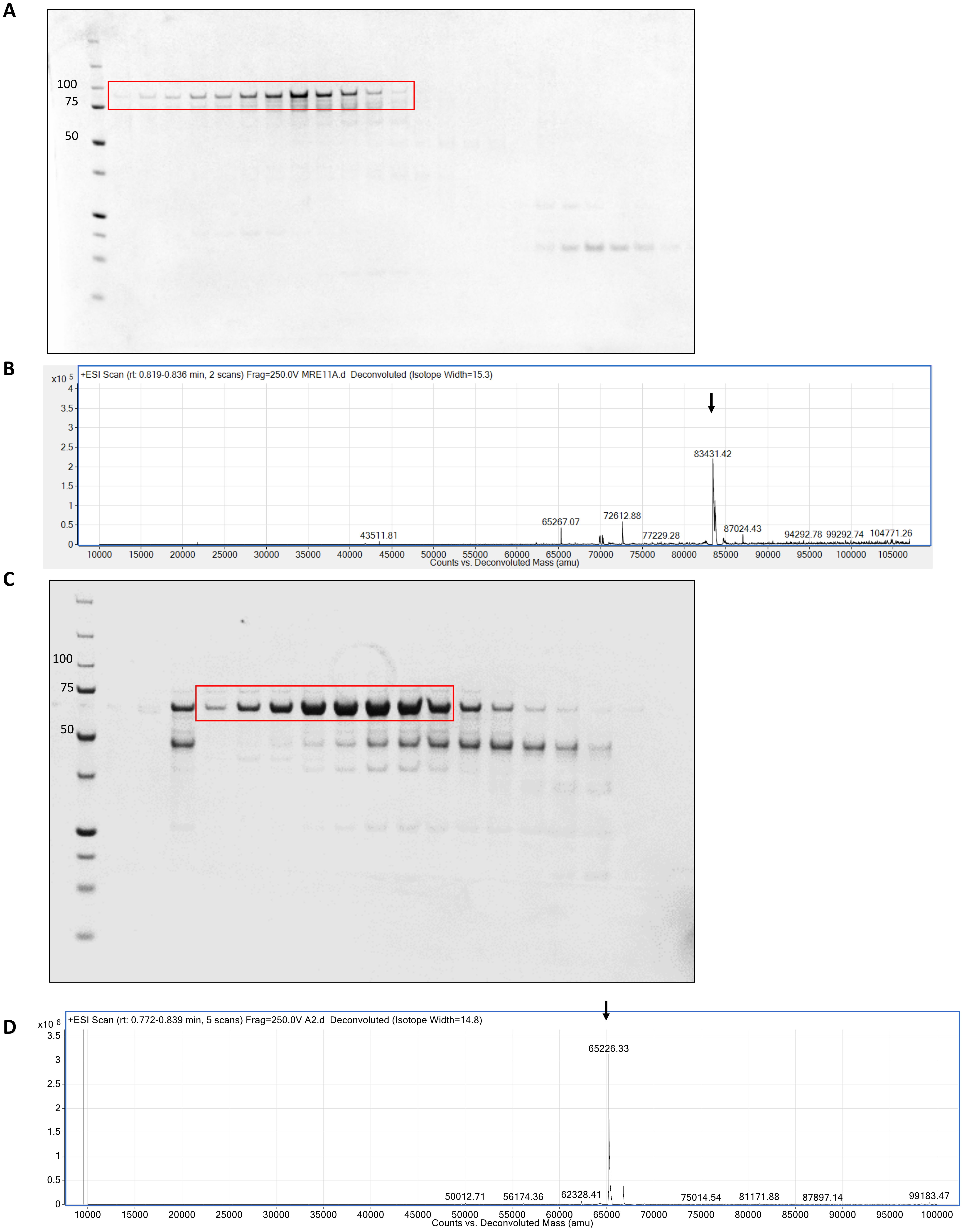

Figure S4 (related to Figure 3). FL and TR-MRE11 recombinant protein purification. (A) Coomassie stained SDS page gel of FL-MRE11 after gel filtration. (B) Intact MS result of FL-MRE11. The major peak at 83431 Da corresponds to the expected mass of 83428 Da for MRE11, including the N-terminal His tag and TEV cleavage site. (C) Coomassie stained SDS page gel of TR-MRE11 after gel filtration. (D) Intact MS result of TR-MRE11. The major peak at 65226 Da correspond to expected mass 65207 Da for TR-MRE11 residues 1-568 aa.

Figure S5 (related to Figure 4)

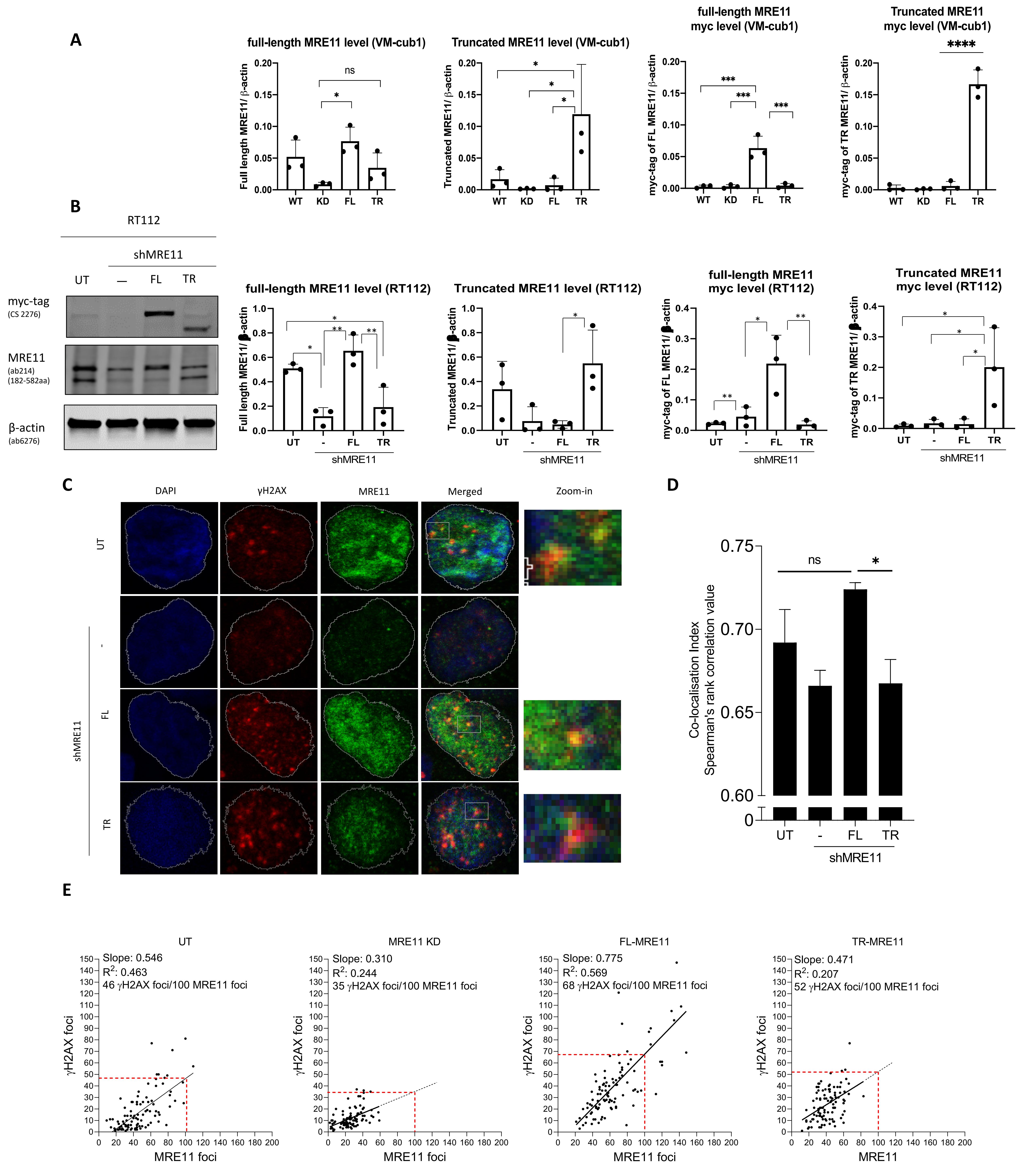

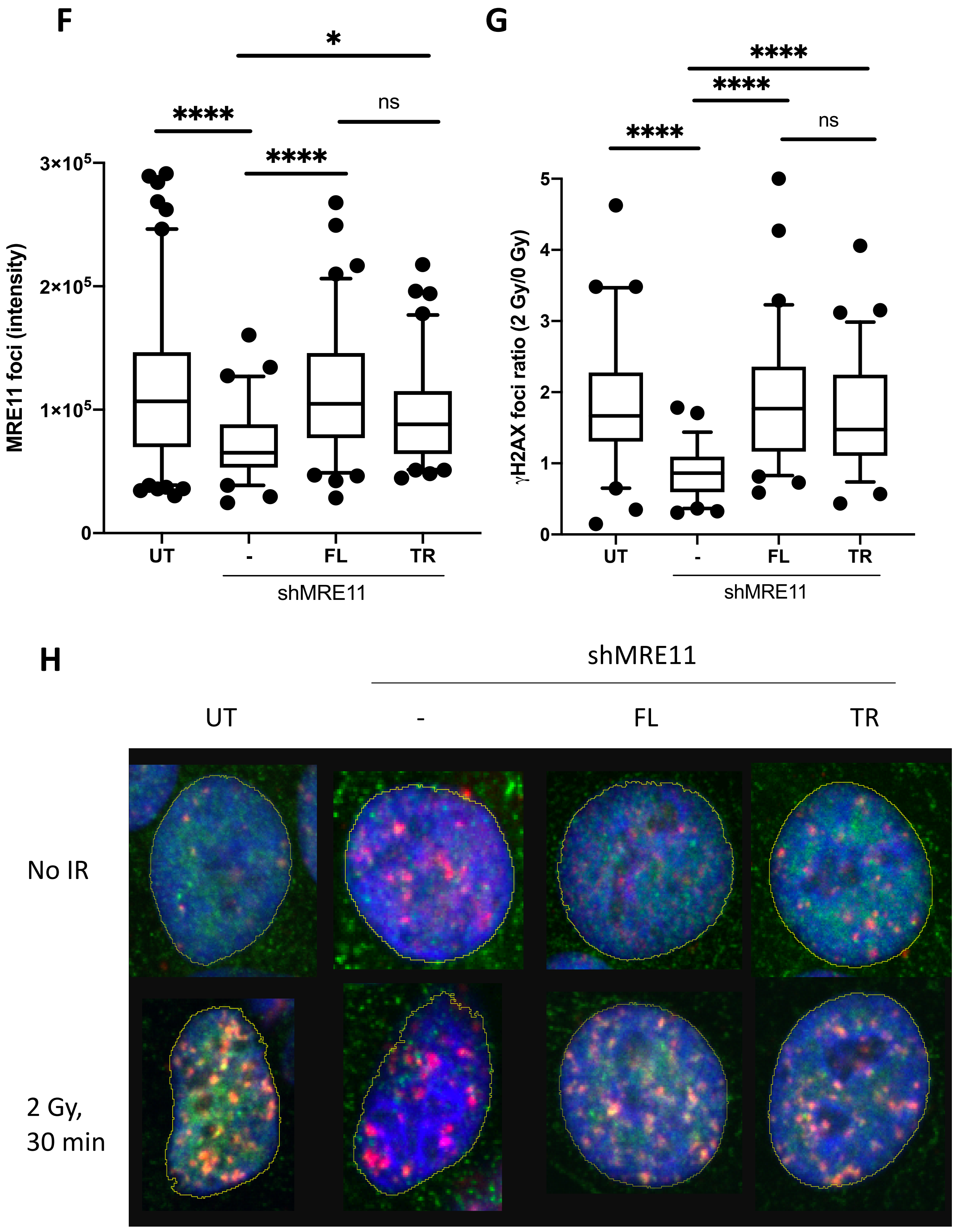

Figure S5 (related to Figure 4). TR-MRE11 co-localises less frequently with γH2AX than FL-MRE11 after IR. (A) MRE11 and myc-tag expression of Fig 4A were confirmed 3 different times after several passages from VM-CUB1 cell lines. (B) Western blot of established RT112 cell lines. Endogenous MRE11 was knocked down and replaced with either exogenous FL or TR-MRE11, and named MRE11 knockdown (KD), FL-MRE11, and TR-MRE11 cells, respectively. MRE11 expression and myc-tag expression were checked regularly to avoid using cell line which has difference in transgene expression. (C) Immunofluorescence microscopy of MRE11 and γH2AX foci showing co-localisation and some failure of co-localisation after radiation (2 Gy, 24 hr post-IR). It also shows that clear knocked down level of MRE11 in RT112 cell lines. (D) TR-MRE11 colocalises less with γH2AX than FL-MRE11 does in RT112 cell lines. (E) The correlation between MRE11 and γH2AX foci numbers from (C). MRE11 deficiency and TR-MRE11 cells showed relatively lower degree of slope for γH2AX foci ratio compare to high expression of MRE11. Data are presented as means $\pm$SEM ($P < 0.0001$ (****), $P < 0.001$ (***), $P < 0.01$ (**), $P < 0.05$ (*); one-way ANOVA, n=3). (F) MRE11 foci levels by immunofluorescence in RT112. FL and TR-MRE11 rescued cells show higher MRE11 intensity than MRE11 KD. (G) The ratio (2 Gy/0 Gy) of γH2AX foci at 30 min post-IR in RT112 cells. The γH2AX foci level increased for all groups except the MRE11 knockdown cell line. The difference in number of foci between FL-MRE11 and TR-MRE11 was non-significant at 30 min. (H) Representative images for MRE11 and γH2AX foci from IF (30 min, 2 Gy).

Figure S6 (related to Figure 5)

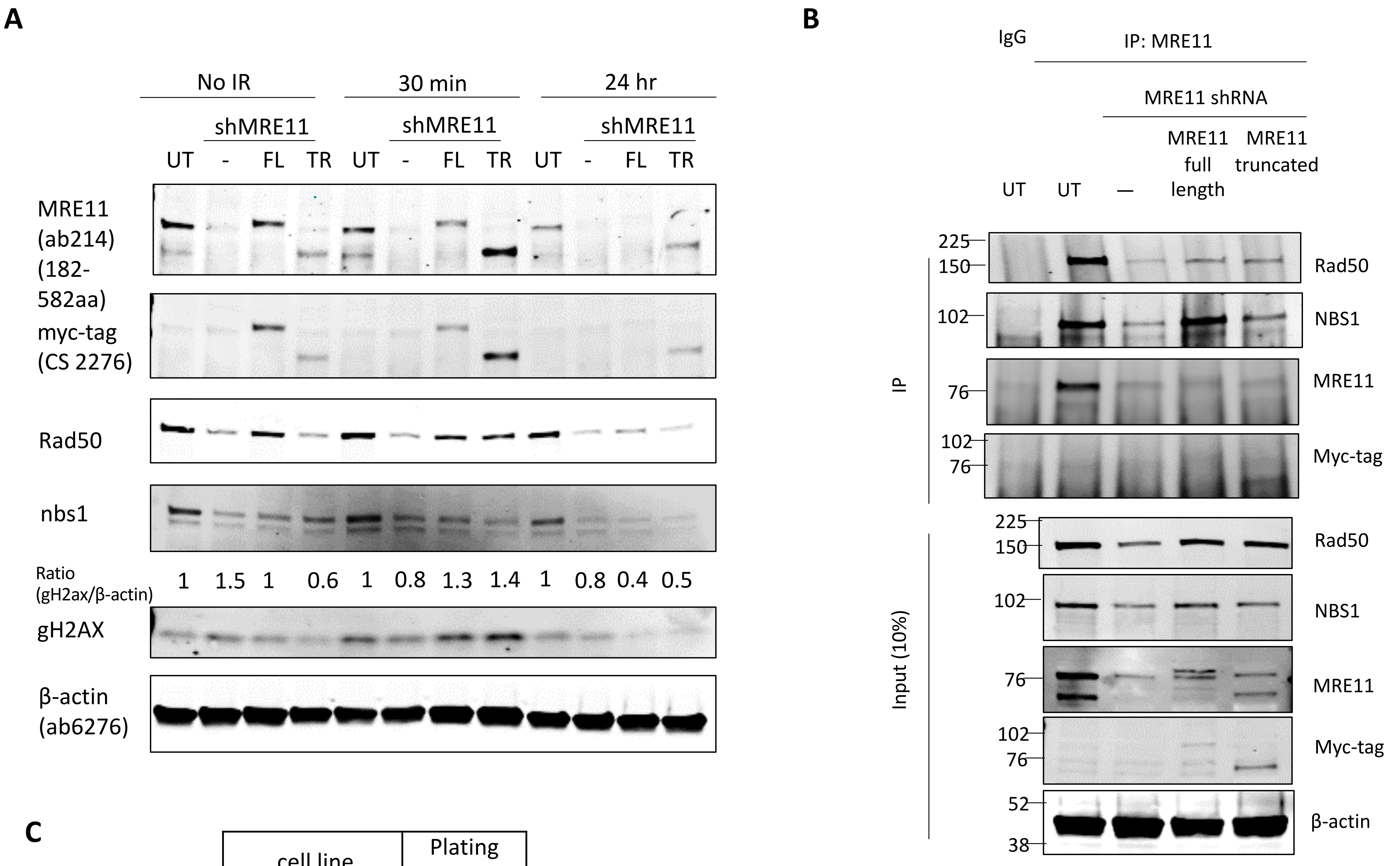

C

cell line		Plating efficiency
VM-CUB1	WT	50.89%
	KD	56.69%
	FL	59.66%
	TR	55.17%
RT112	WT	29.08%
	KD	30.15%
	FL	30.50%
	TR	38.83%

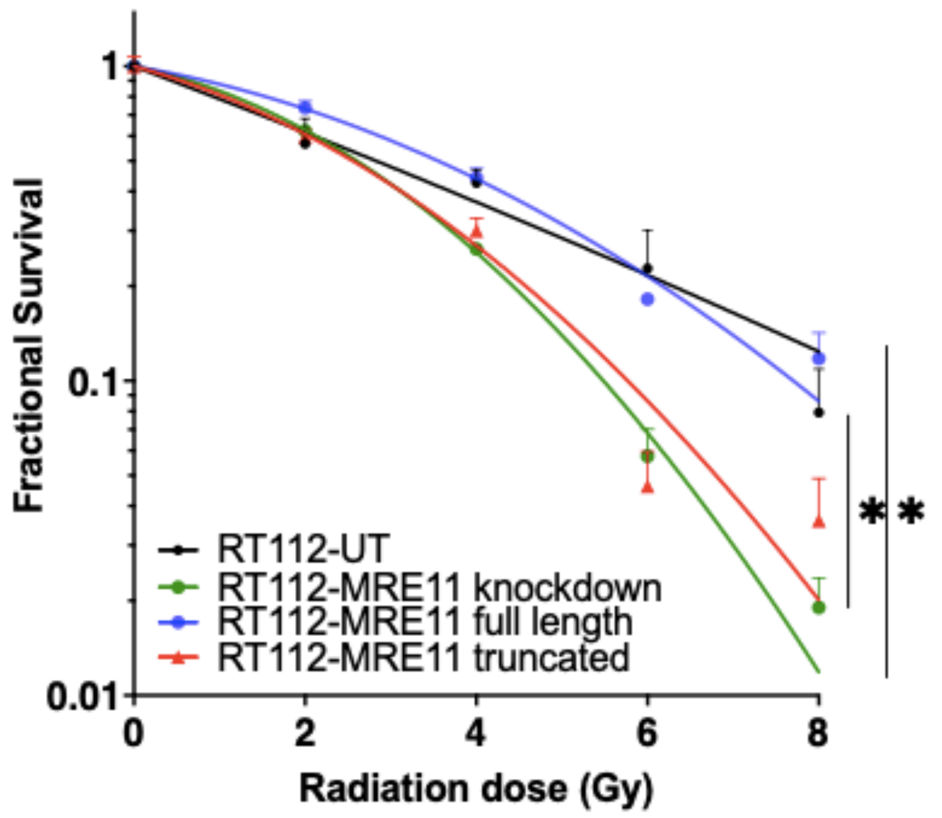

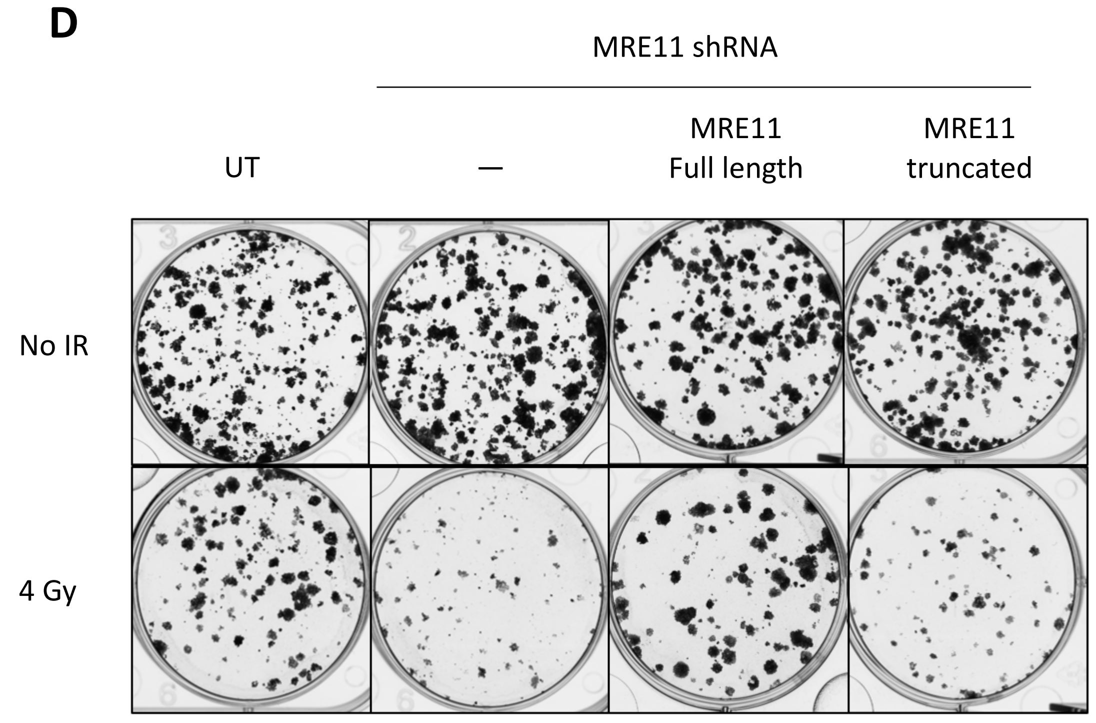

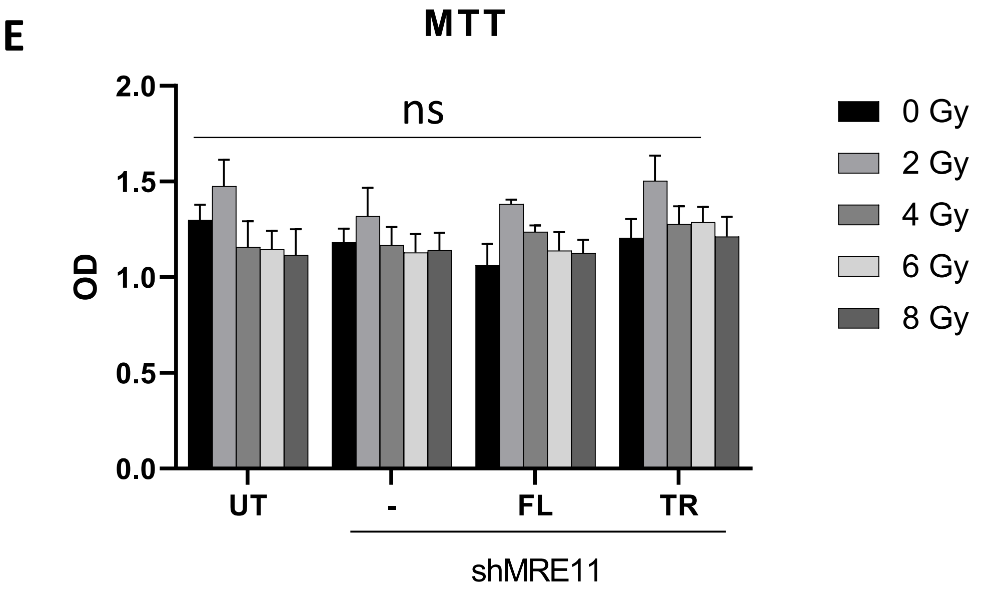

Figure S6 (related to Figure 5). RT112 cells expressing TR-MRE11 are more radiosensitive and deficiency in HR repair than FL-MRE11 expressing cells. (A) The protein expression levels of MRE11 and myc-tag were unchanged after IR (2 Gy) except FL-MRE11 at 24 hr. γH2AX, RAD50 and NBS1 level of FL-MRE11 at 24 hr were extremely low, presumably because of lack of FL-MRE11 as MRE11 knockdown. The other patterns of expression remained the same as Figure 5A. (B) TR-MRE11 was still able to efficiently interact with RAD50 and NBS1, using immunoprecipitated MRE11. (C) Clonogenic survival of TR-MRE11 cells was significantly lower than that of FL-MRE11 and had a significance ($P<0.05$). Also, KD cells had lower survival than UT cells ($P<0.05$). The plating efficiency for each cell line is noted in the table. (D) Representative image of clonogenic plates in (C). (E) MTT assay after treating various with dose of IR showed no significant differences between cell lines.

Figure S7 (related to Figure 6)

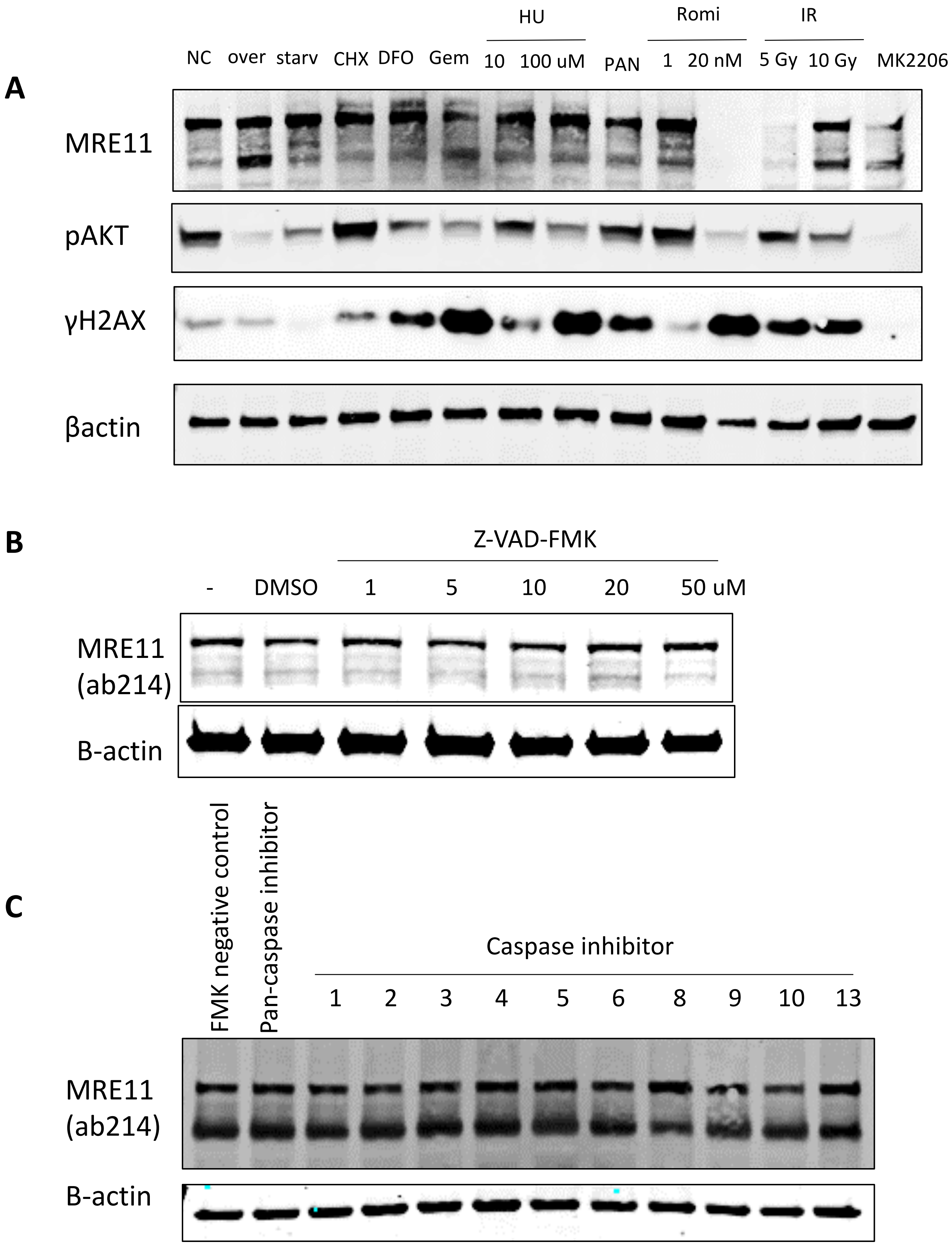

Figure S7 (related to Figure 6). MRE11 expression is unchanged following cells' exposure to various forms of DNA damages and caspase inhibition. (A) MRE11 levels upon various types of damages. These included blocking translational elongation, creation of new DNA, histone deacetylase inhibition and caspase inhibition (cycloheximide (CHX) 100 µM, Deferoxamine mesylate (DFO) 100 µM, Gemcitabine (Gem) 100 µM, Hydroxyurea (HU) 10 or 100 µM, Panobinostat (PAN) 25 nM, Romidepsin (Romi) 1 or 20 nM, MK2206 2 µM, treated for 2 days for all conditions). (B) Treatment of RT112 bladder cancer cells with increasing doses of the pan-caspase inhibitor, Z-VAD-FMK did not alter MRE11 expression levels. (C) Treatment of RT112 bladder cancer cells treated with the individual caspase inhibitors 1, 2, 3, 4, 5, 6, 8, 9, 10, and 13, did not alter MRE11 expression levels.

Figure S8 (related to Figure 6)

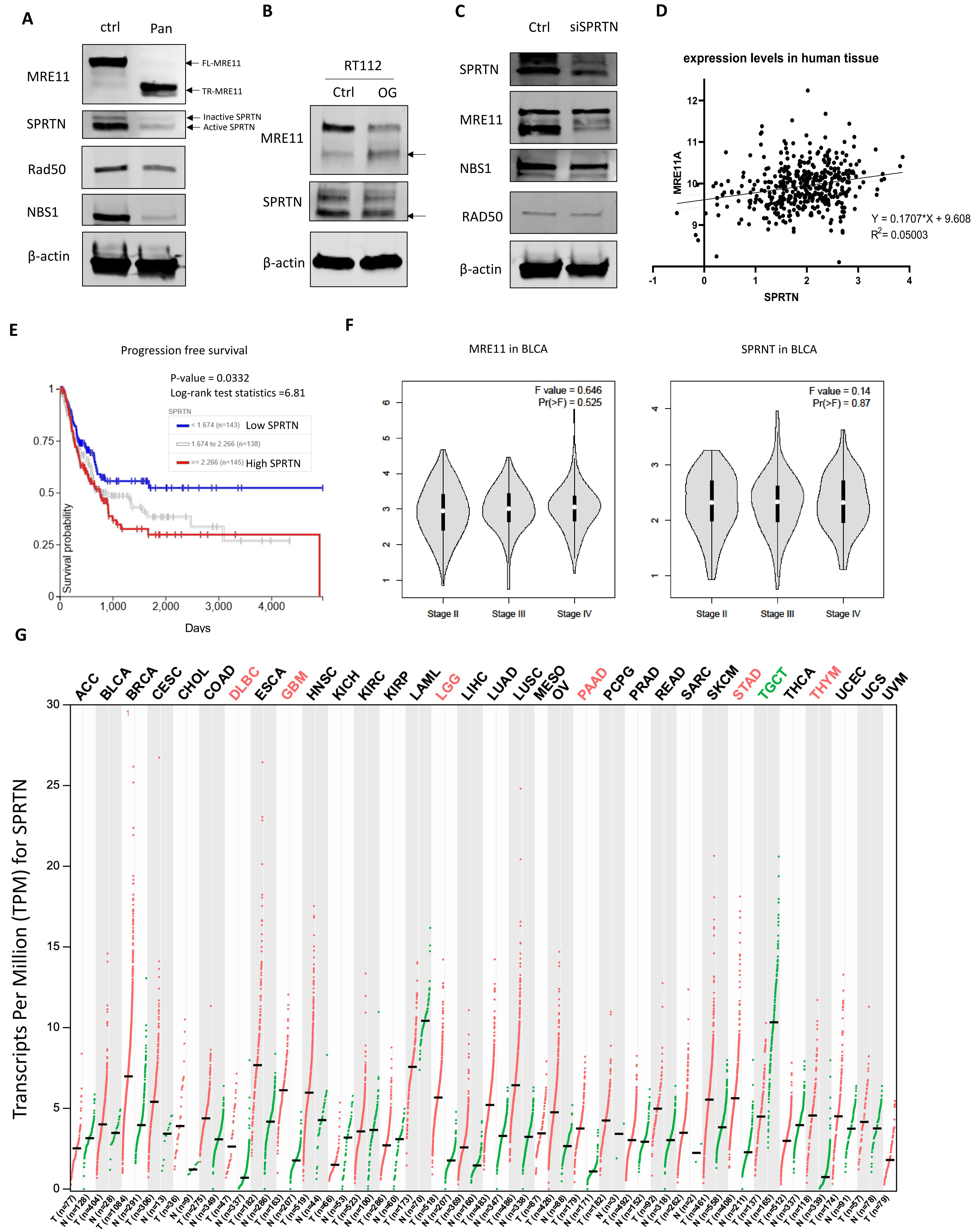

Figure S8 (related to Figure 6). Active SPRTN was potentially consumed to produce TR-MRE11, and higher SPRTN levels were observed the poorer prognosis group from TCGA data. (A) Full-length MRE11 was cleaved after Panobinostat (10 nM, for 24 hr) treatment, and produced truncated MRE11 forms in T24 cells. The active SPRTN level also decreased after Panobinostat treatment. Both RAD50 and NBS1 showed decreased expression after Panobinostat treatment. (B) Full-length MRE11 was cleaved after overgrowth and produced higher levels of truncated MRE11 forms in RT112 cells. The active SPRTN level remained unchanged. (C) siRNA-mediated knockdown of SPRTN (100 nM) decreased TR-MRE11 level. MRE11 interacting partners such as NBS1 and RAD50 maintained the same expression level. (D-G) TCGA analysis was performed for SPRTN and MRE11 expression level in both normal and tumour samples. (D) The correlation between SPRTN and MRE11 levels was not statistically significantly different. (E) The group of patients expressing lower SPRTN had a higher progression-free survival rate. (F) Both MRE11 and SPRTN expression levels in tumours were not dependent on clinical stage. (G) SPRTN levels in both normal and tumour samples were compared for various types of tissue. Most did not show any significant changes in SPRTN level but SPRTN levels were elevated in several tumour samples, including Lymphoid Neoplasm Diffuse Large B-cell Lymphoma (DLBC), Glioblastoma multiforme (GBM), Brain Lower Grade Glioma (LGG), Pancreatic adenocarcinoma (PAAD), Stomach adenocarcinoma (STAD), and Thymoma (THYM) (Red colour represents increased SPRTN level in tumours). Testicular Germ Cell Tumour (TGCT) is the only tumour type which showed downregulation of SPRTN compared to normal tissue (Green coloured). All TCGA data were acquired from https://ucsc-xena.gitbook.io.

Abbreviations: Adrenocortical carcinoma (ACC), Bladder Urothelial Carcinoma (BLCA), Breast invasive carcinoma (BRCA), Cervical squamous cell carcinoma and endocervical adenocarcinoma (CESC), Cholangio carcinoma (CHOL), Colon adenocarcinoma (COAD), Esophageal carcinoma (ESCA), Head and Neck squamous cell carcinoma (HNSC), Kidney Chromophobe (KICH), Kidney renal clear cell carcinoma (KIRC), Kidney renal papillary cell carcinoma (KIRP), Acute Myeloid Leukemia (LAML), Liver hepatocellular carcinoma (LIHC), Lung adenocarcinoma (LUAD), Lung squamous cell carcinoma (LUSC), Mesothelioma (MESO), Ovarian serous cystadenocarcinoma (OV), Pheochromocytoma and Paraganglioma (PCPG), Prostate adenocarcinoma (PRAD), Rectum adenocarcinoma (READ), Sarcoma (SARC), Skin Cutaneous Melanoma (SKCM), Thyroid carcinoma (THCA), Uterine Corpus Endometrial Carcinoma (UCEC), Uterine Carcinosarcoma (UCS), and Uveal Melanoma (UVM).
